# Augmented epigenetic repression of hepatitis B virus covalently closed circular DNA by interferon-α and small-interfering RNA synergy

**DOI:** 10.1128/mbio.02415-24

**Published:** 2024-11-21

**Authors:** Kongying Hu, Wenjing Zai, Mingzhu Xu, Haiyu Wang, Xinluo Song, Chao Huang, Jiangxia Liu, Juan Chen, Qiang Deng, Zhenghong Yuan, Jieliang Chen

**Affiliations:** 1 Key Laboratory of Medical Molecular Virology (MOE/NHC/CAMS), Research Unit of Cure of Chronic Hepatitis B Virus Infection (CAMS), Shanghai Frontiers Science Center of Pathogenic Microbes and Infection, School of Basic Medical Sciences, Shanghai Medical College Fudan University, Shanghai, China; 2 Shanghai Institute of Infectious Disease and Biosecurity, Shanghai, China; 3 Key Laboratory of Molecular Biology of Infectious Diseases (MOE), Chongqing Medical University, Chongqing, China; Virginia Polytechnic Institute and State University, Blacksburg, Virginia, USA; Penn State College of Medicine, Hershey, Pennsylvania, USA

**Keywords:** HBV, cccDNA, IFN, siRNA, epigenetic modulation, combination therapy

## Abstract

The persistence of hepatitis B virus (HBV) covalently closed circular DNA (cccDNA) is a key obstacle for HBV cure. This study aims to comprehensively assess the effect of interferon (IFN) and small-interfering RNA (siRNA) combination on the cccDNA minichromosome. Utilizing both cell and mouse cccDNA models, we compared the inhibitory effects of IFNα, siRNA, and their combination on cccDNA activity and assessed its epigenetic state. IFNα2 treatment alone reduced HBV RNAs, HBeAg, and HBsAg levels by approximately 50%, accompanied by a low-level reconstitution of SMC5/6—a chromatin modulator that restricts cccDNA transcription. HBx-targeting siRNA (siHBx) achieved significant suppression of viral antigens and reconstitution of SMC5/6, but this effect could be reversed by the deacetylase inhibitor Belinostat. The combination of IFN with siHBx resulted in over 95% suppression of virological markers, reduction in epigenetic activation modifications (H3Ac and H4Ac) on cccDNA, and further reduced cccDNA accessibility, with the effect not reversible by Belinostat. In an extracellular humanized IFNAR C57BL/6 mouse model harboring recombinant cccDNA, the effect of combination of clinically used pegylated IFNα2 and GalNac-siHBx was further clarified, indicating a higher and more durable suppression of cccDNA activity compared to either therapy alone. In conclusion, the combination of IFNα and siRNA achieves a more potent and durable epigenetic inhibition of cccDNA activity in cell and mouse models, compared to monotherapy. These findings deepen the understanding of cccDNA modulation and strengthen the scientific basis for the potential of combination therapy.

**IMPORTANCE:**

Since there are currently no approved drugs targeting and silencing covalently closed circular DNA (cccDNA), achieving a “functional cure” remains difficult. This study aims to comprehensively compare the effects of IFNα, small-interfering RNA targeting hepatitis B virus (HBV), and their combination on the activity, accessibility, and epigenetic modifications of cccDNA minichromosomes in cell models. A more durable and stable inhibition of HBV RNAs and antigens expression by IFNα and HBx-targeting siRNA (siHBx) synergy was observed, associated with augmented epigenetic repression of the cccDNA minichromosome. Besides, in an extracellular humanized IFNAR mouse model harboring recombinant cccDNA with an intact response to human IFNα, the synergistic effect of clinically used pegylated IFNα2 and in-house-developed GalNac-siHBx was further clarified.

## INTRODUCTION

Chronic hepatitis B infection (CHB) can lead to hepatitis, cirrhosis, and potentially liver cancer, posing a serious threat to public health (1). It is estimated that 290 million individuals are currently chronic hepatitis B virus (HBV) carriers worldwide (2). As a hepatotropic DNA virus, HBV has a unique replication cycle. The HBV virion contains a genome in relaxed circular DNA (rcDNA) form, which, upon infecting liver cells, is transported to the nucleus and converted into covalently closed circular DNA (cccDNA). cccDNA serves as the template for HBV transcription and replication, producing four major classes of HBV RNAs: the 3.5 kb preC/pre-genomic (pg) RNA, the 2.4 kb and 2.1 kb viral RNAs encoding large, medium, and small envelope proteins (L/M/S HBsAg), and the 0.7 kb RNA encoding the HBV non-structural protein HBx. Among these, pgRNA also serves as a template for HBV reverse transcriptional replication, with most newly replicated rcDNA further secreted in the Dane particle as a fully infectious virion and others replenishing the nuclear cccDNA pool (3, 4). Studies have shown that cccDNA forms a minichromosome in the nucleus, with its transcriptional activity regulated by host epigenetic mechanisms, including changes in chromosomal accessibility and epigenetic modifications. A research on liver biopsy tissues from CHB patients confirmed that the acetylation states of histones H3 and H4 on cccDNA are positively correlated with HBV transcriptional activity, and exogenous use of acetylase inhibitors can effectively inhibit HBV transcription (5). Chromatin immunoprecipitation (ChIP) technology has identified various host transcriptional factors, epigenetic modulators, and viral factors, including HBc and HBx, bound to the cccDNA minichromosome (5–8). Structural Maintenance of Chromosomes (SMC) complexes are key regulators of chromosome dynamics in eukaryotic cells, and studies indicate that the SMC5/6 complex inhibits HBV cccDNA transcription (9), while the HBx protein plays a crucial role in promoting cccDNA minichromosome transcription (10, 11). The persistent presence and transcription of cccDNA are key obstacle for a CHB cure.

Currently, the drugs used clinically to treat CHB mainly include interferon-α (IFNα) and nucleos(t)ide analogs (NAs). IFNα has antiviral and immunomodulatory effects, with advantages such as a finite course of treatment and relatively high rates of HBeAg/HBsAg serum conversion, but the overall response and cure rates remain significantly limited, and there is a risk of side effects (12, 13). NAs act by inhibiting the HBV reverse transcription step, but, since they do not directly target cccDNA, they seldom lead to HBV eradication, thus requiring long-term or even lifelong therapy, and relapse is common after cessation. The concept of “functional cure for CHB” has been proposed in recent years: that is, persistent negativity for HBV DNA and HBsAg in the blood, with cccDNA in the liver in a long-term inactive transcriptional state (14). However, since there are currently no approved drugs targeting cccDNA, achieving a “functional cure” is still quite difficult.

Due to the limitations of existing drugs, various novel anti-HBV candidate drugs have emerged in recent years, among which small-interfering RNA (siRNA) targeting HBV RNAs is considered a complementary new antiviral approach (15, 16). Clinical trial data have shown that it can potently suppress viral antigen expression (17). The HBV genome has overlapping open reading frames with a common poly A sites, allowing a single siRNA to target multiple HBV RNA transcripts. HBx-targeting siRNA (siHBx) can degrade HBx RNA and indirectly affect cccDNA activity by reducing HBx and subsequent reappearance of SMC5/6, in addition to degrading other HBV RNAs with overlapping sequences (18). However, HBV siRNA candidates still face issues such as incomplete suppression of HBV antigens and relapse. On the other hand, IFNα can promote the degradation and clearance of HBV RNAs at the post-transcriptional level and reduce antigen production by epigenetically regulating the cccDNA minichromosome (19, 20), thereby lowering its accessibility and transcriptional activity. Given the complementary mechanisms of IFN and siRNA, the combination of IFN and HBV siRNA could be more effective in clearing viral antigens and enhancing the sustained response after cessation, as evidenced by several clinical trials (21). However, whether this combination could exert additive to synergistic anti-viral effects and achieve a higher and more durable silencing of cccDNA remains unclear.

To this end, this study aims to comprehensively compare the effects of IFNα, siRNA targeting HBV, and their combination on the activity, accessibility, and epigenetic modifications of cccDNA minichromosomes in cell models. Besides, in an extracellular humanized IFNAR (IFNAR-hEC) mouse model harboring recombinant cccDNA with an intact response to human IFNα, the effect of combination of clinically-applied pegylated IFNα2 and in-house developed GalNac-siHBx was further clarified.

## MATERIALS AND METHODS

### Cell culture and HBV infection

The HepG2^NTCP^ cell line (kindly provided by Prof. Stephan Urban) and HepG2 were cultured as we previously described (22). Production of the virions from HepAD38 cell line (23), HBV infection, and HBVcircle transfection was performed as described (24). HepAD38 was cultured in a medium without DOX for 7–10 days to allow HBV replication before treatment. HepDES19 is a HepG2-derived cell line with a 1.1-fold mutant S gene integrated into the HBV genomeD under tet-off regulation, which is provided by Prof. Jutao Guo (25). This mutant cell is unable to translate and form the HBV S protein, nor can it secrete intact viral particles. Consequently, most of the core particles in the cytoplasm will retrograde the rcDNA back into the nucleus to replenish and form new cccDNA. When using this cellular model for experimental studies on cccDNA-related functions, the cells are pre-cultured in a medium without DOX for 7–10 days to allow cccDNA accumulation. HepG2-HBV/*loxp* was established in-house (26). It is a HepG2-derived cell line with a single copy of the HBV genomeD integrated, which contains loxP sites. By transducing the cell with an Ad-Cre recombinant adenovirus, the Cre enzyme can recognize and cleave the loxP sites at both ends of the HBV genome, releasing the linear HBV genome from the integration site and facilitating the recombination into rcccDNA.

### Plasmids, antibodies, primers, and reagents

The antibodies, siRNAs, and primers used in this study are listed in Tables S1, S2, and S3, respectively. All plasmids were prepared using NucleoBond Xtra Midi EF (MACHEREY-NAGEL). HBVcircle was generated in-house as described (27). Belinostat (PXD101) was purchased from MedChemExpress. The levels of HBeAg and HBsAg in the cell culture supernatant or mouse serum were determined by ELISA (AutoBio, China). The antibodies against HBx and core were developed in-house by immunizing mice with HBx-derived peptides or core proteins. The levels of HBV DNA in the cell culture supernatant or mouse serum were determined by detection Kit (Sansure, China). Total RNA was purified from TRIzol Reagent extraction (Ambion), followed by Northern Blot analysis, RNA sequencing (RNA-seq), or reverse-transcription to complementary DNA using PrimeScript RT reagent Kit (TaKaRa). Quantitative PCR (qPCR) was performed using TB Green Premix Ex Taq (TaKaRa) in a StepOne Real-Time PCR System. Cells were lysed in RIPA lysis buffer and then quantified by BCA Protein Quantification Kit (Vazyme), followed by western blot analysis. siHBx, siHBc, and siHBs in this study, the non-targeting control siRNA (siNC), the siRNA targeting host apolipoprotein B (ApoB; siApoB), and the fluorescein (FITC)-labeled siRNAs were all synthesized by RIBOBIO Company (Guangzhou). For *in vivo* studies, siHBx was further chemically modified and conjugated with N-acetyl-D-galactosamine (GalNac-siHBx) to increase stability and liver uptake.

### Hirt DNA extraction and detection of HBV cccDNA

HBV cccDNA was extracted from HBV-infected cells by a modified Hirt method. Briefly, take a six-well plate for example, cells were submerged in 500 µL of buffer I (50 mM Tris pH 7.5, 10 mM EDTA, 50 µg/mL RNase A; frozen mouse liver specimens were thawed and ground in buffer I) and lysed by the addition of 500 µL of 1.2% g/mL SDS at room temperature for 15 min. Cellular debris and chromosomal DNA were precipitated by the addition of 700 µL of buffer III (3M CsCl, 1M potassium acetate, 0.67M acetic acid). The tubes were gently mixed and placed in 4°C for 2 hours. After centrifugation, the supernatant was loaded onto a silica gel membrane column (QIAprep Spin Miniprep Kit). Then the column was washed and eluted by centrifugation. The HBV cccDNA in modified Hirt DNA was either quantified by qPCR with specific primers or detected by Southern blot analysis (24).

### Chromatin immunoprecipitation

For cell samples, ChIP experiments were carried out on infected cells 9 days post-infection. Briefly, cells were fixed with 1% formaldehyde for 10 min at room temperature and quenched with 0.125 M Glycine. For nuclear extracts preparation, cells were lysed in cell lysis buffer (10 mM HEPES, pH7.9, 0.5% IGEPAL-CA630, 1.5 mM MgCl2, 10 mM KCl, and EDTA-free protease inhibitors [Roche]). After centrifugation, nuclei were lysed in nuclei lysis buffer (50 mM Tris, pH 8.1, 10 mM EDTA, 0.3% SDS, and EDTA-free protease inhibitors). After sonication, lysates were diluted 1:3 with dilution buffer (0.01% SDS, 1% Triton X-100, 1.2 mM of EDTA, 16.7 mM Tris pH8, 167 mM NaCl, and EDTA-free protease inhibitors). Chromatin was then subjected to overnight immunoprecipitation at 4°C using 6 µL of antibodies. Negative controls with nonspecific immunoglobulins were included in each experiment. Immune complexes were incubated with protein A/protein G agarose beads at 4°C, washed, and eluted in elution buffer (20 mM Tris–HCl, 1 mM EDTA, pH 8, 0.4% SDS, and 0.2 µg/µL). Immunoprecipitated DNA was quantified by qPCR using cccDNA-specific primers. Samples were normalized to input DNA using the ∆Ct method, where ∆Ct = Ct (input) − Ct (immunoprecipitation), and calculated as percentage of the input. Results were presented as the average of at least three independent experiments. ChIP experiments were also carried out on mouse liver samples. The frozen specimens were thawed and cut into pieces, followed by fixing with 1% formaldehyde for 10 min at room temperature and quenching with 0.125 M Glycine. After centrifugation, pieces of tissue were ground into single-cell suspension in cell lysis buffer and then were lysed. The following steps were the same as those for cell samples.

### Assay for transposase-accessible chromatin and the next-generation sequencing

The assay for transposase-accessible chromatin (ATAC) sequencing (ATAC-seq) was performed using TruePrep DNA Library Prep Kit V2 for Illumina (Vazyme, China) according to the manufacturer’s instructions, and the next-generation sequencing (NGS) was done by Annoroad Gene Technology (Beijing, China) using the Illumina NGS platforms.

### The recombinant cccDNA-based IFNAR-hEC mouse model

The IFNAR-hEC C57BL/6 mouse model was established in-house, featuring an intact response to human-type I IFNs, including the currently clinically used PEG-IFNα2 (28). The recombinant AAV-rcccDNA and AAV-Cre viruses were constructed in-house, and equal amounts of rAAV-rcccDNA and rAAV-Cre viruses (2 × 10^10^ viral genome [v.g.] copies per mice per virus) were intravenously administered to the mice via tail vein, thereby generating recombinant cccDNA in the liver (29). After 2–4 weeks of injection, animals were bled 1 day before the start of treatment and grouped to obtain similar HBsAg and HBeAg levels. For siRNA treatment, GalNac-siHBx was subcutaneously injected at a dose of 2.5 mg/kg at monthly dose frequency (two doses in total). For IFN treatment, PEG-IFNα2 (trade name: Pegasys, Roche) was administered subcutaneously at twice per week dose frequency and at a dose of 25 ng/g body weight (12 doses in total). The mice were either sacrificed 6 hours after the last injection of PEG-IFNα or left untreated for follow-up analysis.

All animal studies were conducted in accordance with the guidelines for the care and use of medical laboratory animals and approved by the Animal Ethics Committee of Fudan University. Mice were bled by retro-orbital puncture at indicated time points. Specimens from mice were either frozen at −80°C or 4% formaldehyde fixed for immunohistochemistry and hematoxylin and eosin (H&E) staining.

### Statistical analysis

All the data sets from cell experiments are based on a minimum of three independent experiments. Data were analyzed and presented using GraphPad Prism 8.0. For comparison of two datasets, data were analyzed using Student’s *t* test or two-way analysis of variance (ANOVA) with Sidak multiple comparison correction. The data were presented as the mean ± SD. Statistical significance was considered with a *P* value less than 0.05 (*P < 0.05; **P < 0.01; ***P < 0.001).

## RESULTS

### The combination of IFNα and siHBx more effectively and durably suppresses HBV antigen and RNA production in HBV-infected cells

**Fig 1:**
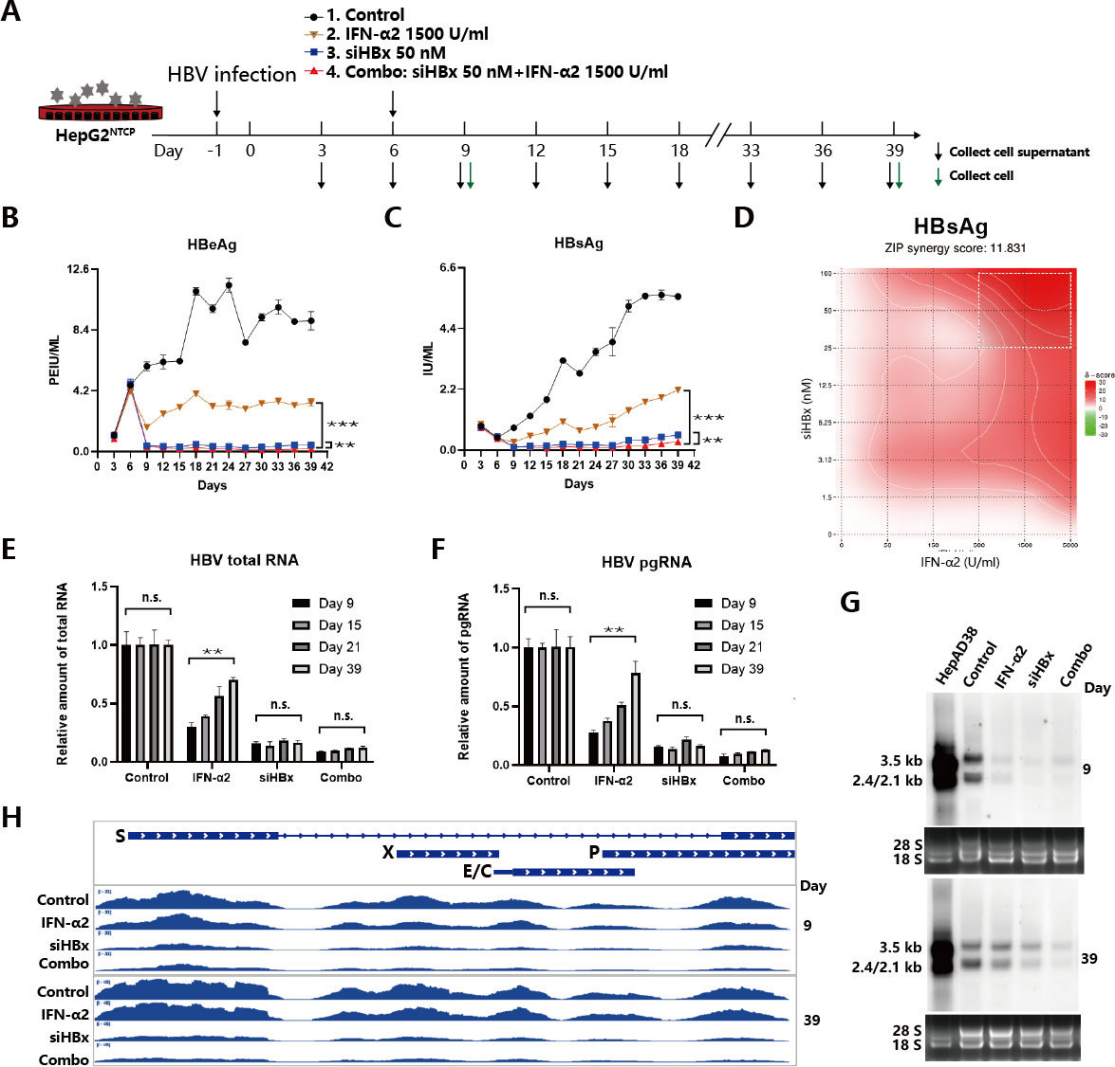
Antiviral effects of IFNα2, siHBx, and their combination in HBV-infected HepaG2^NTCP^ cells. (**A**) Schematic view of experiment. HepG2^NTCP^ cells were infected with HBV at MOI = 800. One day later, cells were washed and cultured with DMEM supplemented with 10% FBS and 2.5% DMSO. Six days post-infection (dpi), cells were treated as follows: the control group was transfected with siNC (50 nM), the IFNα2 group was treated one-time with IFNα2 (1,500 U/mL), the siHBx group was transfected one-time with siHBx (50 nM), and the combination (combo) group was treated with transfection of siHBx (50 nM) followed by IFNα2 (1,500 U/mL) treatment. Cell supernatants and cells were collected at indicated time points for subsequent analysis. (**B**) The kinetics of HBeAg and (**C**) HBsAg levels in the supernatants at indicated time points were displayed. (**D**) HBV-infected HepG2^NTCP^ cells were treated with varying concentrations of siHBx and IFNα2 at 6 dpi. HBsAg levels in the supernatants at 9 dpi were determined by ELISA. The inhibition rates of HBsAg were calculated and imported into the software SynergyFinder. (**E**) Intracellular HBV total RNA and (**F**) pgRNA relative levels at different time points were determined via qPCR analysis with specific primers. (**G**) Intracellular HBV RNA levels at 9 or 39 dpi were determined via northern blot analysis. Total RNA of Dox-off HepAD38 cells were used as markers. (**H**) Intracellular total RNAs were extracted and applied for RNA-seq analysis. HBV RNA reads were visualized via IGV. Data were analyzed by unpaired two-tailed Student’s *t* tests and presented as means ± SD.

**Fig 2:**
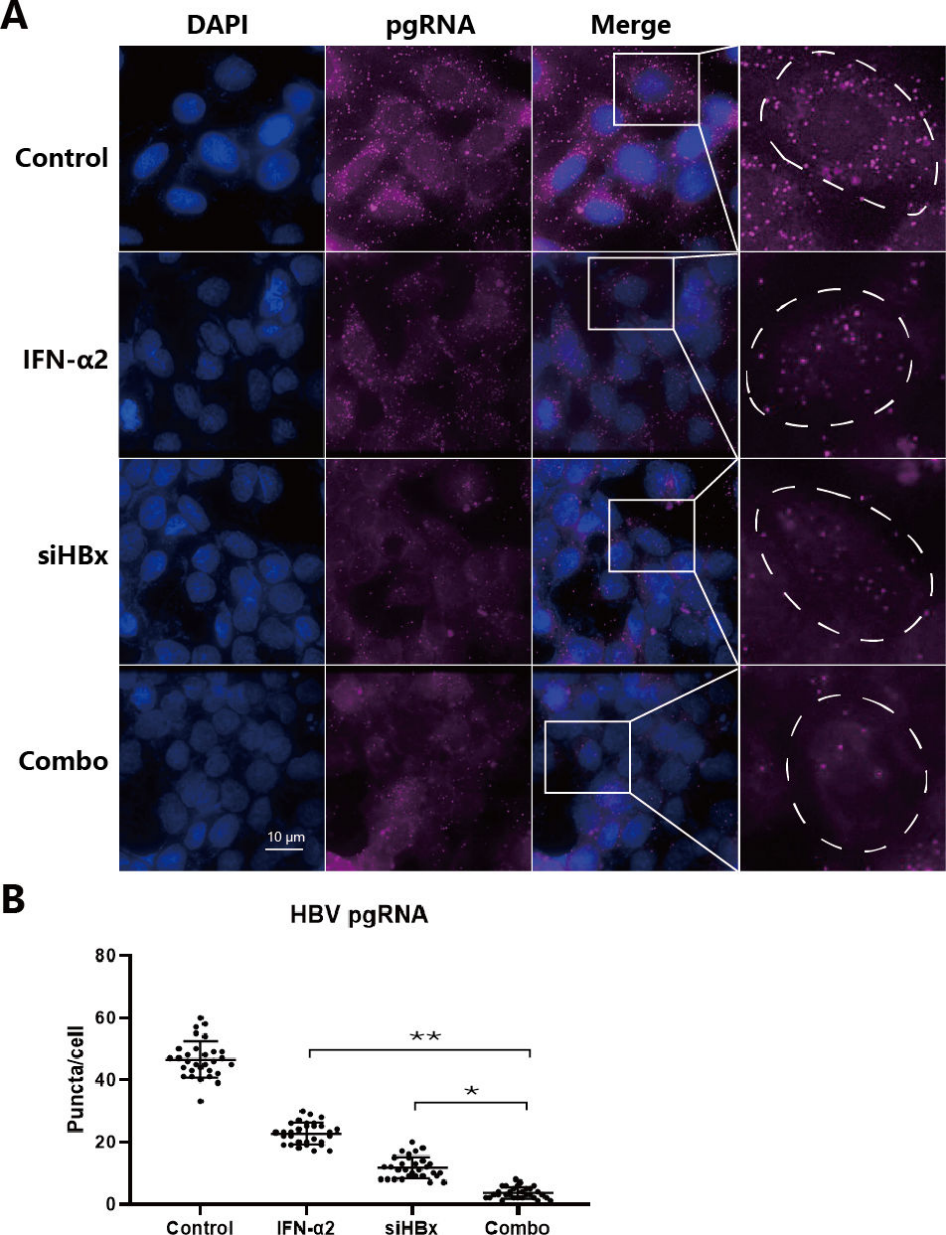
Detection of HBV pgRNAs with specific probe in HepAD38 cells. (**A**) HepAD38 cells were mono- or combo-treated with siHBx (50 nM) and IFNα2 (1,500 U/mL) for 2 days, after which the pgRNA signals were tested by FISH. Scale bar indicates 10 µm. (**B**) Quantitative analysis of punctate fluorescent signals of pgRNA. Thirty cells per group were counted. Data were analyzed by unpaired two-tailed Student’s *t* tests and presented as means ± SD.

To compare the suppressive effects of IFNα, siHBx, and their combination on HBV antigens and RNAs, we first used the HBV-infected HepG2^NTCP^ cell model. After confirming successful HBV infection based on antigen levels (Fig. S1A and B), on day 6, cells were divided into four groups for treatment: the control group, transfected with siNC; the group received a single treatment of IFNα2 at a concentration of 1,500 U/mL; the group received a single treatment of siHBx transfected at a concentration of 50 nM; the combination treatment group, first transfected with siHBx, then after 6 hours and medium change, IFNα2 was added (Fig. 1A). HBeAg and HBsAg levels in the cell supernatants were measured using ELISA. HBV DNA levels in the cell supernatants were measured using qPCR. The results indicated that compared to the control group, IFNα alone reduced HBeAg, HBsAg, and HBV DNA levels by about 70%, 60%, and 20% (Fig S2A and B), respectively, and exhibited varying degrees of rebound over time (Fig. 1B and C); siHBx alone reduced them by about 85%, 85%, and 40%, respectively (Fig. 1B and C; Fig S2A and B); while the combination treatment reduced them by about 95%, 90%, and 60%, respectively (Fig. 1B and C; Fig S2A and B). With the concentration of siHBx reduced (25, 12.5, and 6.25 nM), the difference between the combination group and the siHBx alone group became more significant (Fig. S3A and B), further clarifying the role of IFNα2. Additionally, treatment with IFNα2 and siHBx showed synergistic effects in inhibiting HBsAg (Fig. 1D) and HBeAg (Fig. S3C) in HBV-infected HepG2^NTCP^ cells, though the magnitude of this effect was modest, as determined by the ZIP synergy scores >10 (12.063). The white rectangle indicates the region of the maximum synergistic area. These results suggest that compared to using IFNα2 or siHBx treatment alone, their combination further suppresses HBV antigen expression and maintains the durability of suppression. The results of analyzing the expression of HBV RNAs showed that in the IFNα2 group, the relative levels of HBV total RNA and pgRNA gradually rebounded, while their expression remained at about 90% and 95% inhibition levels in the siHBx group and the combination group (Fig. 1E and F). Northern blot analysis further confirmed the changes in HBV RNAs upon different treatments, and there was some rebound in HBV RNA levels in the siHBx or IFNα2 mono-treatment groups by day 39, while the repression of HBV RNA in the combination group remained (Fig. 1G). Further RNA-seq analysis showed that the reads of HBV RNAs in the combination group at days 9 and 39 were at extremely low levels; whereas, there was a certain degree of rebound in HBV sequence reads in the IFNα2 group by day 39 (Fig. 1H). Furthermore, both pre- or simultaneously incubating MycludexB (MyB), an HBV entry inhibitor, with HBV-blocked HBV infection (Fig. S4A and B). However, there was no influence on HBeAg and HBsAg levels when MyB was given at 6 and 9 days post-infection (Fig. S4C and D). These results indicated that *de novo* infection rarely occurs in these HBV-infected cells; the observed inhibitory effects of IFNα, siHBx, and their combination are primarily due to the inhibition of cccDNA transcription. These results indicate that in HBV-infected HepG2^NTCP^ cells, the combination of siHBx with IFNα2 can achieve a higher-level suppression of HBV antigen and RNA expression, and a single administration can result in a relatively durable inhibition. We also confirmed that the combination of IFNα2 and siHBx can inhibit HBV antigens and RNAs at a higher level compared with IFNα2 or siHBx treatment alone in the HBV replicating model HepAD38 (Fig. S5) and cccDNA models (HepG2-HBV/loxp and HepDES19; Fig. S6). In HepAD38 cells, we used the FISH assay to statistically analyze the number of pgRNA at the single-cell level in different treatment groups: approximately 45 puncta in the control group, while in the IFNα2 or siHBx treatment alone groups, the puncta were reduced to 10–20, and in the combination group, they further decreased to about 4 (Fig. 2). In addition, the concentration of IFNα2 (1,500 U/mL) or siRNA (50 nM) used in the study has no cytotoxic effect (Fig. S7).

As the inhibitory effect of siHBx mono-treatment on HBV antigen and RNA expression seems also relatively sustained, we examined whether the siRNAs could persist within the cells for a prolonged period by using FITC-labeled siRNA (siRNA-FITC). At 24 and 48 hours post transfection of HepG2^NTCP^ cells with siRNA-FITC, obvious green fluorescent spots could be observed. As time goes by, at 72 and 120 hours, almost no green fluorescent spots were observed (Fig. S8A), suggesting that the presence of siRNA after transfection into cells may be relatively short-lived, suggesting the sustained inhibitory effect of siHBx on HBV antigen and RNA may not be due to the continuous presence of siRNA. Moreover, we designed siRNA targeting the liver-specific protein apolipoprotein B (APoB; siAPoB) and transfected to HepG2^NTCP^ cells. The results showed that the expression levels of *APoB* mRNA were relatively low at days 2 and 9 post transfection, but with prolonged time, their expression levels gradually recovered to normal levels (Fig. S8B), further supporting the short lifespan of siRNA and its related direct effect on mRNA degradation. Furthermore, we investigated whether the trans-complementation of HBx after siHBx transfection could rescue cccDNA activity. HBeAg and HBsAg secretion were rescued when HBx was trans-complemented by Lenti-HBx (Fig. S8C).

### IFNα and siHBx do not directly degrade HBV cccDNA

**Fig 3:**
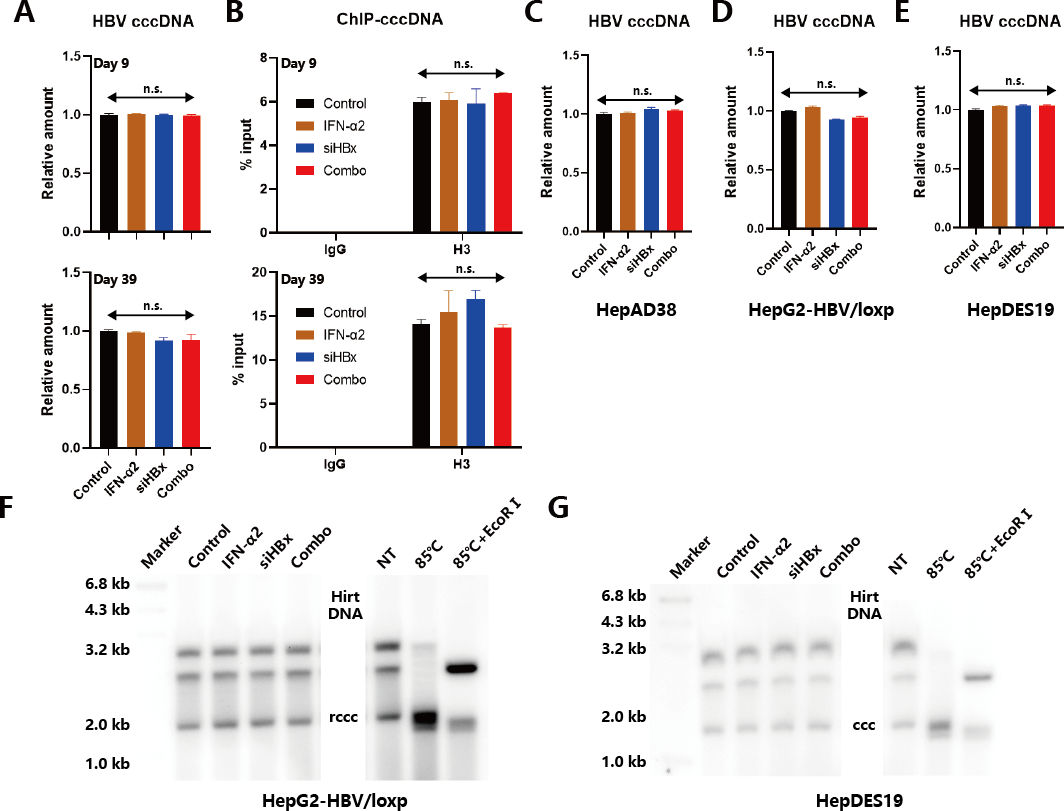
The effects of IFNα2, siHBx, and their combination on the quantity of cccDNA. (**A**) HepG2^NTCP^ cells were infected with HBV and were divided into four groups as described in Fig. 1A. Intracellular cccDNA relative levels at 9 and 39 dpi were determined via qPCR analysis with specific primers. (**B**) The levels of histone H3 bound to cccDNA in different groups at 9 and 39 dpi were determined via ChIP-qPCR analysis with specific antibodies and primers. (**C**) HepAD38, (**D**) HepG2-HBV/loxp, and (**E**) HepDES19 were treated with IFNα2 (1,500 U/mL), siHBx (50 nM), or both of them. After 2 days, intracellular cccDNA was extracted via Hirt method and detected by qPCR. (**F**) HepG2-HBV/loxp and (**G**) HepDES19 were treated with IFNα2 (1,500 U/mL), siHBx (50 nM), or both of them. After 2 days, intracellular cccDNA was extracted via Hirt method and detected by Southern blot analysis. Data were analyzed by two-way ANOVA with Sidak multiple comparison correction and presented as means ± SD.

We next investigated whether the suppressive effect on HBV antigens by IFNα and siHBx is achieved through direct clearance of cccDNA or not. We extracted HBV cccDNA using the Hirt method, then used qPCR to detect the relative quantity of cccDNA. Results showed that in HBV-infected HepG2^NTCP^ cells, there was no statistical difference in the relative quantity of cccDNA between the four groups on both day 9 and day 39 (Fig. 3A; Fig. S9A), indicating that the differences in HBV antigen and RNAs observed between the groups were not due to the differences in the quantity of the cccDNA template within the cells. Next, we used ChIP-qPCR to detect the histone protein bound to cccDNA minichromosomes in HBV-infected HepG2^NTCP^ cells, showing no statistical difference in the relative quantity of histone H3 bound to cccDNA between the four groups on both days 9 and 39 (Fig. 3B). Besides, in HBV replicating model HepAD38 (Fig. 3C) and cccDNA models (HepG2-HBV/loxp and HepDES19; Fig. 3D through G), qPCR and Southern blot results validated that there were no significant differences in cccDNA content between different groups. To verify these results, we further used a HepG2^NTCP^ cell model transfected with HBVcircle and used Southern blot to detect the effect of IFNα2 or siHBx alone and their combination on the quantity of HBVcircle. The results of the HBsAg and HBeAg levels in the supernatants confirmed the anti-HBV effects of IFNα2 and siHBx therapy (Fig. S9B and C). Consistently, no significant differences in HBVcircle content between the four groups were observed in this condition (Fig. S9D). These results indicate that under the working concentrations used in this study, IFNα2, siHBx, and their combination have no direct degrading effect on existing HBV cccDNA within the cells.

### Augmented epigenetic suppression of HBV minichromosomes by IFNα2 and siHBx combination

**Fig 4:**
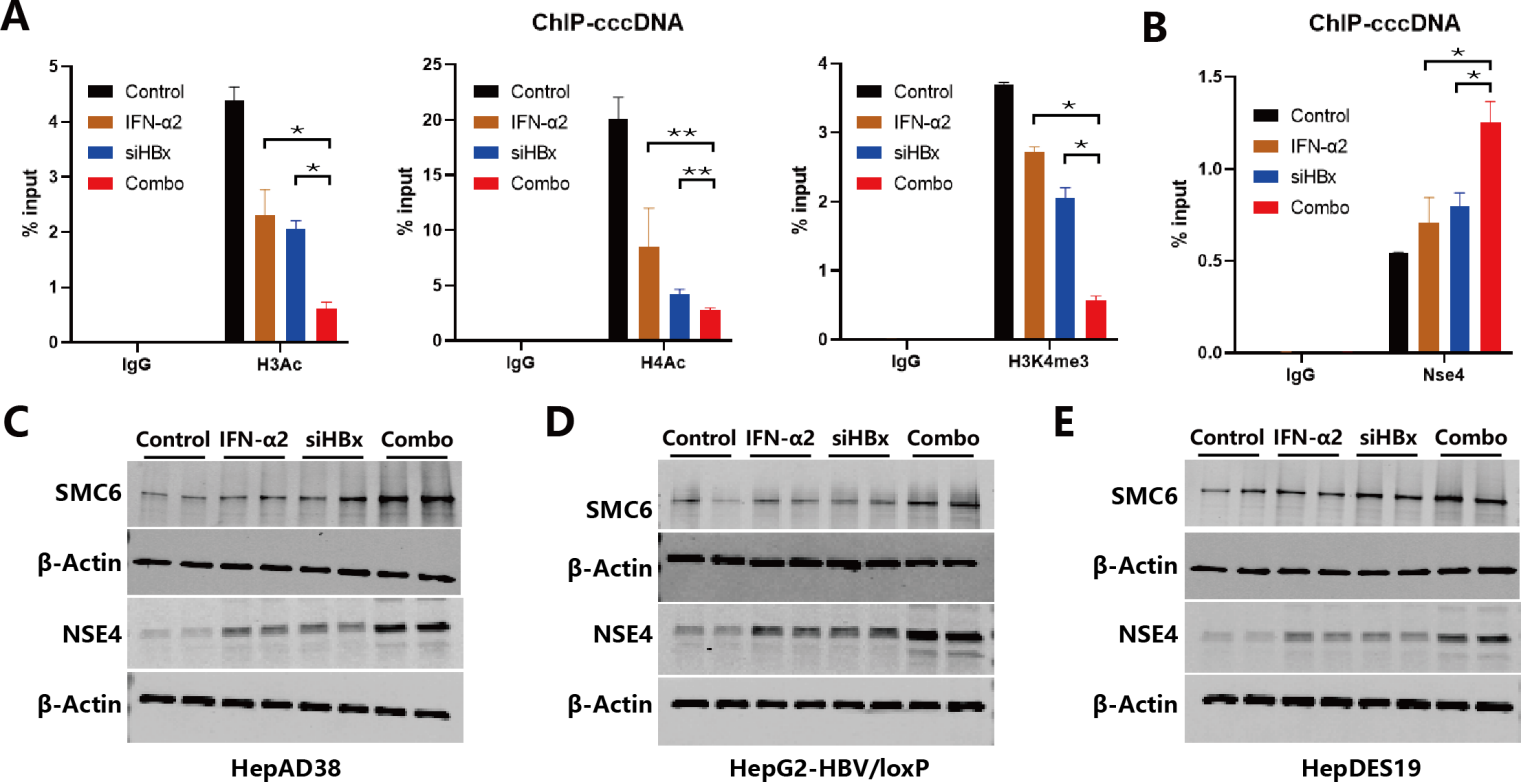
The effects of IFNα2, siHBx, and their combination on the epigenetic modifications and the recruitment of SMC5/6 complexes on cccDNA. (**A**) HepG2^NTCP^ cells were infected with HBV and were divided into four groups as described in Fig. 1A, then the levels of H3Ac, H4Ac, H3K4me3 modifications on cccDNA were determined via ChIP-qPCR analysis at 12 dpi. (**B**) HepG2^NTCP^ cells were infected with HBV and were divided into four groups as described in Fig. 1A, then SMC5/6 complex (Nse4) binding levels on cccDNA were determined via ChIP-qPCR analysis at 12 dpi. (**C**) HepAD38, (**D**) HepG2-HBV/loxp, and (**E**) HepDES19 were treated with IFNα2 (1,500 U/mL), siHBx (50 nM), or both of them. After 2 days, intracellular SMC6 levels were determined via western blot analysis, and β-Actin was applied as loading control. Data were analyzed by unpaired two-tailed Student’s *t* tests and presented as means ± SD.

As the higher level of HBV antigen and RNA suppression under the combination of IFNα2 and siHBx is not achieved through direct clearance of cccDNA, further detection of their effect on cccDNA transcriptional activity was carried out. We thus examined the effect of mono and combination treatments on the representative active epigenetic modifications of cccDNA minichromosomes: H3Ac, H4Ac, and H3K4me3. Results showed that in HBV-infected HepG2^NTCP^ cells, the IFNα2 or siHBx alone groups could only moderately reduce (about 40%–50%) the levels of these epigenetic modifications, while the combination group significantly reduced their levels by about 90% (Fig. 4A), suggesting that the combination of the two has an additive to synergistic suppressive effect on the epigenetic modifications of cccDNA proteins. Given that SMC5/6 plays an important role in cccDNA transcriptional suppression, and HBV can antagonize the effect of SMC5/6 through HBx (9, 11), in the HBV-infected HepG2^NTCP^ model (Fig. 1A), ChIP-qPCR was used to detect the level of the SMC5/6 complex bound to cccDNA minichromosomes (detecting Nse4, which is a representative subunit of the SMC5/6 complex). Results showed that the use of IFNα2 or siHBx alone increased the level of Nse4 bound to cccDNA minichromosomes to about 0.7% of input compared to the untreated control, while their combination increased it to about 1.2% of input (Fig. 4B). We further studied the recovery effect on the host restriction factor SMC5/6. In the HBV replicating cell line HepAD38, it was observed that 1,500 U/mL of IFNα2 and 50 nM of siHBx mono-treatment slightly suppressed HBx expression and recovered SMC5/6, while combo-treatment could significantly suppress HBx expression and recover SMC5/6 as well as NSE4 protein levels (Fig. 4C; Fig. S5C). The same experiment was conducted in HBV cccDNA cell lines HepG2-HBV/loxp and HepDES19, with their recovery effects on the host restriction factor SMC5/6 complexes consistent with those in HepAD38 (Fig. 4D and E). The above results indicate that the combination of IFNα2 and siHBx better enhances the levels of the SMC5/6 complex bound to cccDNA, which can contribute to more effectively reducing the levels of active epigenetic modifications on HBV cccDNA minichromosomes.

**Fig 5:**
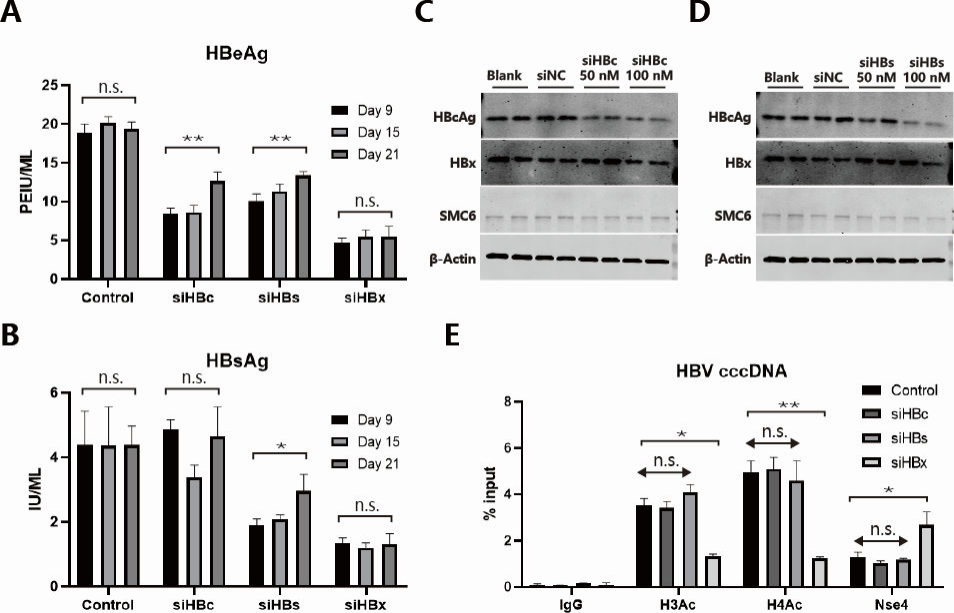
The effects of siHBVs targeting core, HBsAg, and HBx on HBV and SMC5/6 complexes. (**A**) HBV-infected HepG2^NTCP^ cells were transfected with siHBVs targeting core (siHBc), HBsAg (siHBs), and HBx (siHBx) at the concentration of 50 nM at 6 dpi. The HBeAg and (**B**) HBsAg levels in the supernatants at 9, 15, and 21 dpi were determined via ELISA. (**C**) HepAD38 cells were transfected with siHBc (50 and 100 nM) or (**D**) siHBs (50 and 100 nM). After 2 days, intracellular HBcAg, HBx, and SMC6 levels were determined via western blot analysis, and β-Actin was applied as loading control. (**E**) HBV-infected HepG2^NTCP^ cells were transfected with siHBc, siHBs, and siHBx (50 nM) at 6 dpi. The levels of H3Ac, H4Ac modifications, and SMC5/6 complex (Nse4) binding on cccDNA were determined via ChIP-qPCR analysis at 12 dpi. Data were analyzed by unpaired two-tailed Student’s *t* tests or by two-way ANOVA with Sidak multiple comparison correction and presented as means ± SD.

We also compared the suppressive effects of siRNA targeting other HBV RNA segments (including siHBc and siHBs) on HBeAg and HBsAg, as well as their recovery effects on SMC5/6. In the HBV-infected HepG2^NTCP^ cells, using the same system as in Fig. 1A, 3 days after transfection, siHBc could only suppress the expression of HBeAg (suppression level reaching about 50%) but not suppress the expression of HBsAg, while siHBs could suppress the expression of both HBeAg and HBsAg (suppression levels reaching about 40% and 50% respectively), but not as high as the suppression level of siHBx alone (suppression levels reaching about 85% and 85%, respectively; Fig. 5A and B). In addition, HBV-infected HepG2^NTCP^ cells transfected with siHBc and siHBs both showed a trend of HBeAg and HBsAg recovery at day 21. In HepAD38, HepG2-HBV/loxp, and HepDES19 cell lines, 2 days after transfection, compared with siHBc and siHBs, siHBx showed higher inhibition of HBeAg (Fig. S10A, D and G), HBsAg (Fig. S10B and E), and HBV RNAs (Fig. S10C and F). In HepAD38, neither siHBc nor siHBs could recover SMC5/6 (Fig. 5C and D; Fig. S10H). The same experiment was also conducted in HepG2-HBV/loxp and HepDES19, with their recovery effects on the host restriction factor SMC5/6 consistent with those in HepAD38 (Fig. S10I and J). In the HBV-infected HepG2^NTCP^ cells, we also showed that only siHBx can reduce the H3Ac, H4Ac of cccDNA minichromosomes, and recover the binding of Nse4 on cccDNA (Fig. 5E). These results indicate that the efficient suppression of antigens by siHBx is closely related to its targeting of all HBV RNAs including HBx RNA, and its long-lasting suppressive effect could be related to its suppression of HBx expression and thus affecting the epigenetic state of cccDNA.

### The combination of IFNα and siHBx more effectively reduces the accessibility of cccDNA

**Fig 6:**
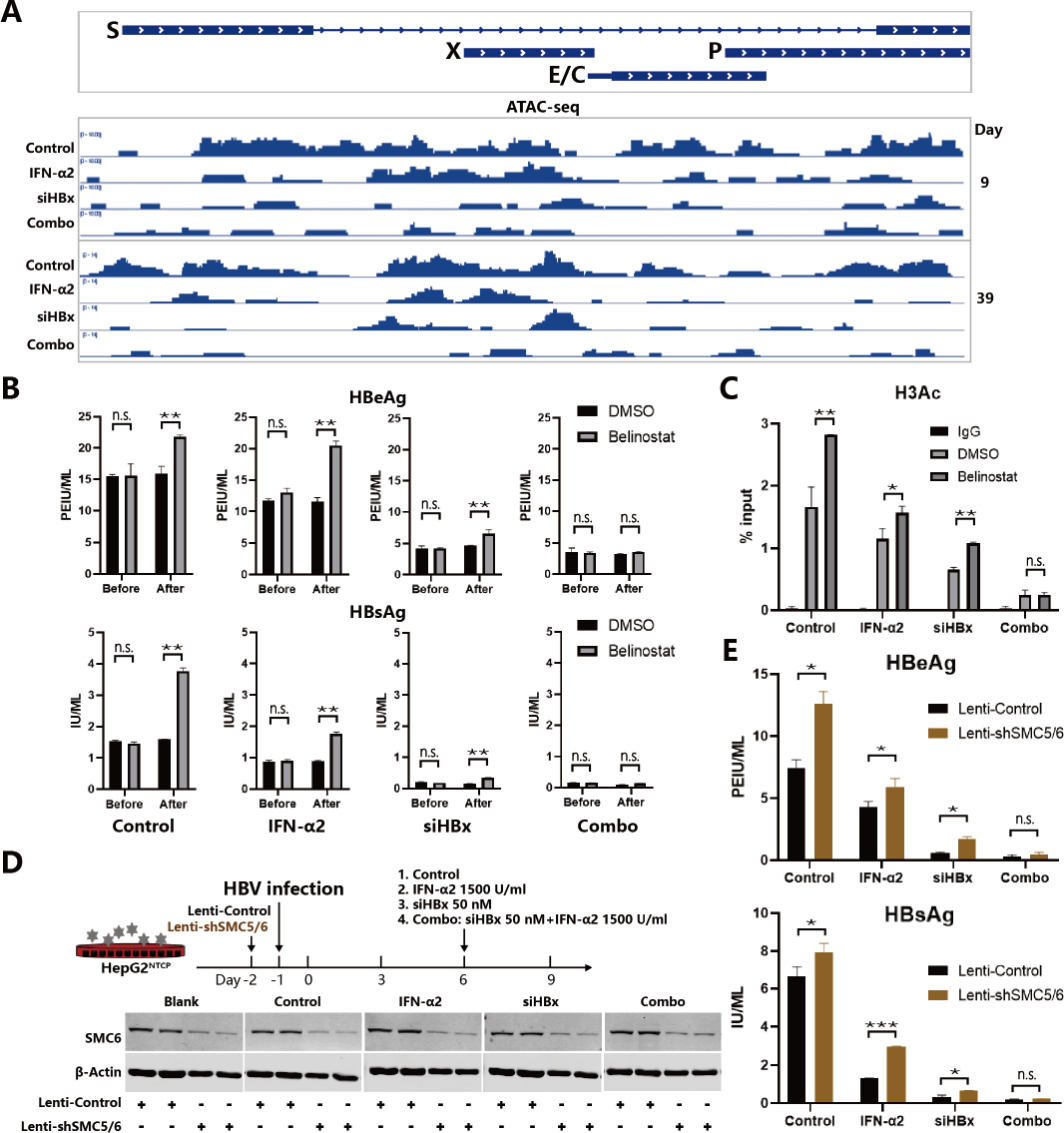
The effects of IFNα2, siHBx, and their combination on the accessibility of cccDNA. (**A**) HepG2^NTCP^ cells were infected with HBV and were divided into four groups as described in Fig. 1A. The accessibility of cccDNA at 9 and 39 dpi was detected by ATAC-seq analysis. (**B**) HepG2^NTCP^ cells were infected with HBV and were divided into four groups as described in Fig. 1A. Cells were treated with Belinostat (40 nM) or DMSO (2.5%) at day 21 dpi. Cell supernatants were collected every 3 days. HBeAg and HBsAg levels 6 days before or after Belinostat and DMSO treatment were determined. (**C**) Cells were treated as described in (**B**). The levels of H3Ac modifications on cccDNA were determined via ChIP-qPCR analysis at day 27 dpi. (**D**) HepG2^NTCP^ cells were transduced with Lenti-shSMC5/6 or Lenti-Control lentivirus 1 day before being infected with HBV and were divided into four groups as described in Fig. 1A. Cell supernatants were collected every 3 days. Intracellular SMC6 levels at day 9 dpi were determined via western blot analysis, and β-Actin was applied as loading control. (**E**) Cells were treated as described in (**D**). HBeAg and HBsAg levels at day 9 dpi in the supernatants were determined via ELISA. Data were analyzed by unpaired two-tailed Student’s *t* tests and presented as means ± SD.

The accessibility of cccDNA is closely related to its activity and regulated by the epigenetic modification state. In the HBV-infected HepG2^NTCP^ model, we used ATAC-seq to detect the accessibility of cccDNA minichromosomes in the four groups on days 9 and 39. Results showed that on day 9, the use of IFNα2 and siHBx alone only partially reduced the accessibility of cccDNA, as indicated by the peaks like those located upstream of E/C open reading frame, while their combination further depressed these peaks and significantly reduced the accessibility of cccDNA; on day 39, the accessibility of cccDNA in the mono-treatment groups showed a rebound trend, while the combination group maintained low accessibility of cccDNA (Fig. 6A). We further used the deacetylase inhibitor Belinostat trying to activate cccDNA in different treatment groups. The results showed that HBsAg and HBeAg levels in the control group, IFNα2 group, and siHBx group all recovered after 6 days of Belinostat treatment, with statistical differences, while the combination group showed no significant change between Belinostat-treated and untreated groups (Fig. 6B). Consistently, ChIP-qPCR results showed that Belinostat further activated the control group, partially recovered the IFNα2 and siHBx groups’ cccDNA minichromosome histone H3Ac modification levels, but did not significantly recover that of the combination group (Fig. 6C). These results indicate that the combination of IFNα2 and siHBx can more effectively suppress the transcriptional activity of cccDNA, and this suppressive effect has a certain stability, which can to some extent resist the epigenetic activation challenge. Given that IFNα2 and siHBx can both promote the binding of the SMC5/6 complex to cccDNA to suppress cccDNA transcription, we then knocked down SMC5/6 in advance on day -2 to observe the effect on HBsAg suppression under different treatments. Results showed that pre-knockdown of SMC5/6 promoted HBeAg and HBsAg expression to varying degrees in the IFNα2 or siHBx treatment groups but had no significant effect on the combination group (Fig. 6D and E), while it did not increase the rates of infection establishment (Fig. S11). The result further suggests that IFNα2 and siHBx can suppress cccDNA transcription to varying degrees, while the combination of IFNα2 and siHBx has an additive to synergistic suppressive effect on cccDNA, which could be partially attributed to the restricted level of SMC5/6.

### PEG-IFNα and GalNAc-siHBx combination more effectively and durably suppress HBV antigens and RNAs in the rcccDNA IFNAR-hEC mouse model

**Fig 7:**
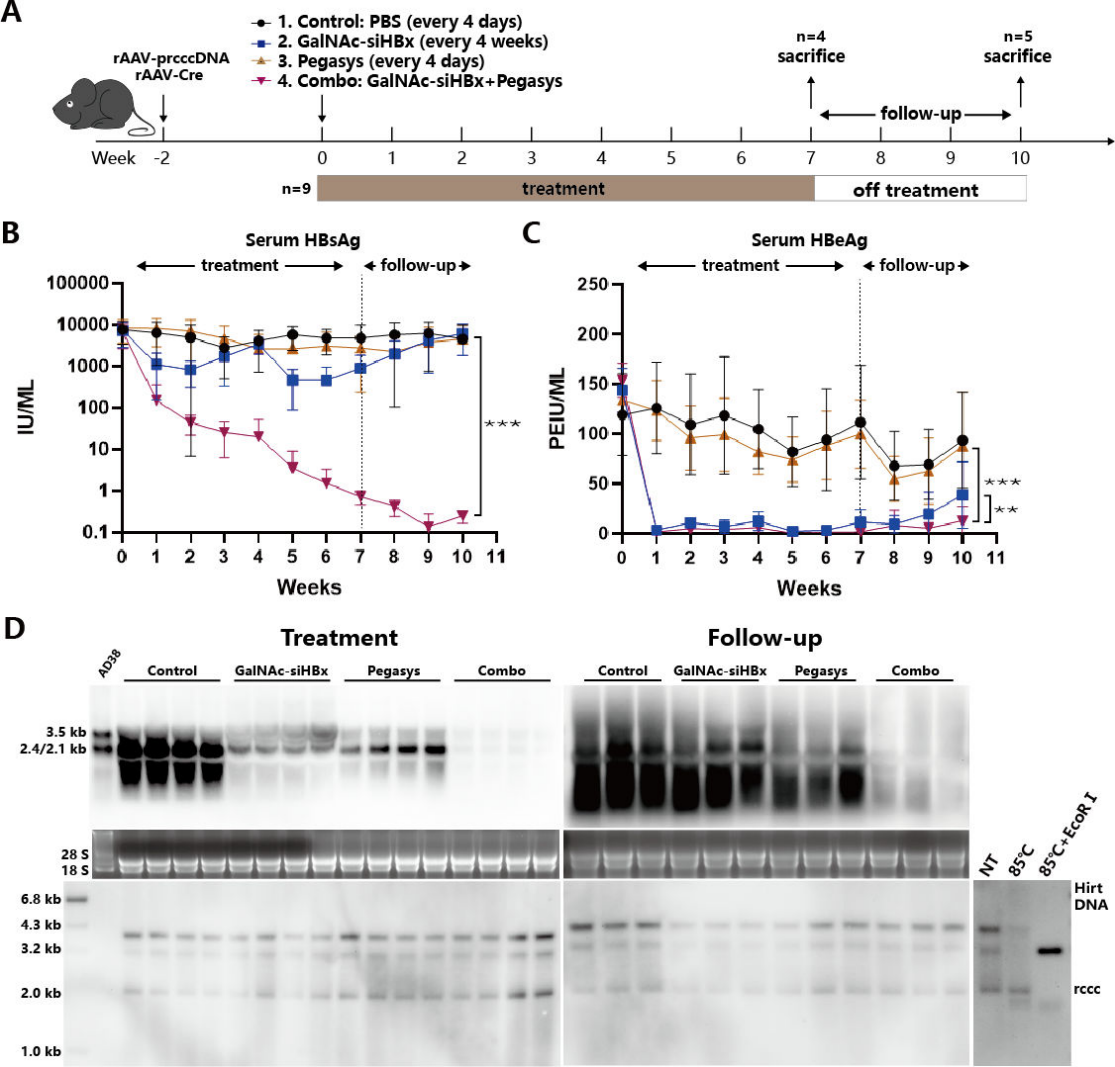
Anti-HBV effects of PEGIFNα2, GalNAc-siHBx, and their combination in rcccDNA IFNAR-hEC mice. (**A**) Schematics of treatment procedures. IFNAR-hEC mice were injected with equal amount of rAAV-rcccDNA and rAAV-Cre viruses (2.5 × 10^10^ v.g per mice) via tail vein 2–4 weeks before bleeding and grouping (*n* = 9 for each group). Mice were treated as follows: the control group was subcutaneously injected with PBS every 4 days (8 mL/kg), the GalNac-siHBx group was subcutaneously injected with GalNac-siHBx once every 4 weeks (2.5 mg/kg, two doses in total), the Pegasys group was subcutaneously injected with Pegasys every 4 days (30 µg/kg, 12 doses in total), and the combination group had the same treatment frequency as the monotherapy groups, with the first administration being a subcutaneous injection of GalNac-siHBx followed by Pegasys 6 hours later. Blood was sampled weekly. Partial mice (*n* = 4 for each group) were stherapyacrificed at the end of IFN and/or siHBx treatment (the “treatment” batch), and the others (*n* = 5 for each group) were left untreated and were continuously monitored for another 3 weeks (the “Follow-up” batch). Six hours before mice were sacrificed, the last dose of Pegasys was subcutaneously injected in Pegasys and combination groups in “treatment” batch. (**B**) Serum HBsAg and (**C**) HBeAg levels were determined via ELISA analysis. (**D**) Intrahepatic HBV RNAs levels were determined via northern blot analysis, and cccDNA was detected via Southern blot analysis. Data were analyzed by unpaired two-tailed Student’s *t* tests and presented as means ± SD.

Based on the *in vitro* observations, we further observe the effect of the combination of IFNα and siRNA on cccDNA *in vivo*. Based on our previously constructed IFNAR-hEC mouse strain which has an intact response to human IFN-I, we established the recombinant cccDNA model (29). Using this model, the clinically applied long-acting interferon (PEG-IFNα2a, trade name Pegasys) can be used for the experiments. We verified in the mouse liver that Pegasys significantly induced the production of various representative interferon-stimulated genes (ISGs; Fig. S12). The mice were divided into four groups: the control group, the Pegasys group, the GalNac-siHBx group, and the combination group (*n* = 9/group). After 7 weeks of treatment, the first batch of mice (*n* = 4/group) from each group was sacrificed. The remaining mice were no longer treated, with blood samples taken weekly to detect serum antigen levels, and they were sacrificed after 3 weeks (Fig. 7A). Results suggest that the serum HBsAg and HBeAg levels in the GalNac-siHBx and Pegasys combination group mice were significantly lower than those in any mono-treatment group, and within 3 weeks after stopping the treatment, the antigen levels in the IFN and siRNA mono-treatment groups gradually recovered, while the combination group maintained low levels (Fig. 7B and C). Southern blot and qPCR results indicate that GalNac-siHBx and Pegasys mono- and combo-treatment had no direct effect on the quantity of rcccDNA in the mouse liver (Fig. 7D; Fig. S13). Northern blot result indicates that 3 weeks after stopping the drug, the HBV RNAs level in the IFN and siRNA mono-treatment groups recovered, while the combination group maintained at low levels (Fig. 7D). These results suggest that the combination of GalNac-siHBx and PEG-IFNα2 can enhance the suppressive effect on HBV RNAs and viral antigens, and this suppressive effect has certain durability after stopping the treatment.

### Augmented epigenetic suppression of HBV minichromosomes by PEG-IFNα and GalNAc-siHBx combination in the rcccDNA IFNAR-hEC mouse model

**Fig 8:**
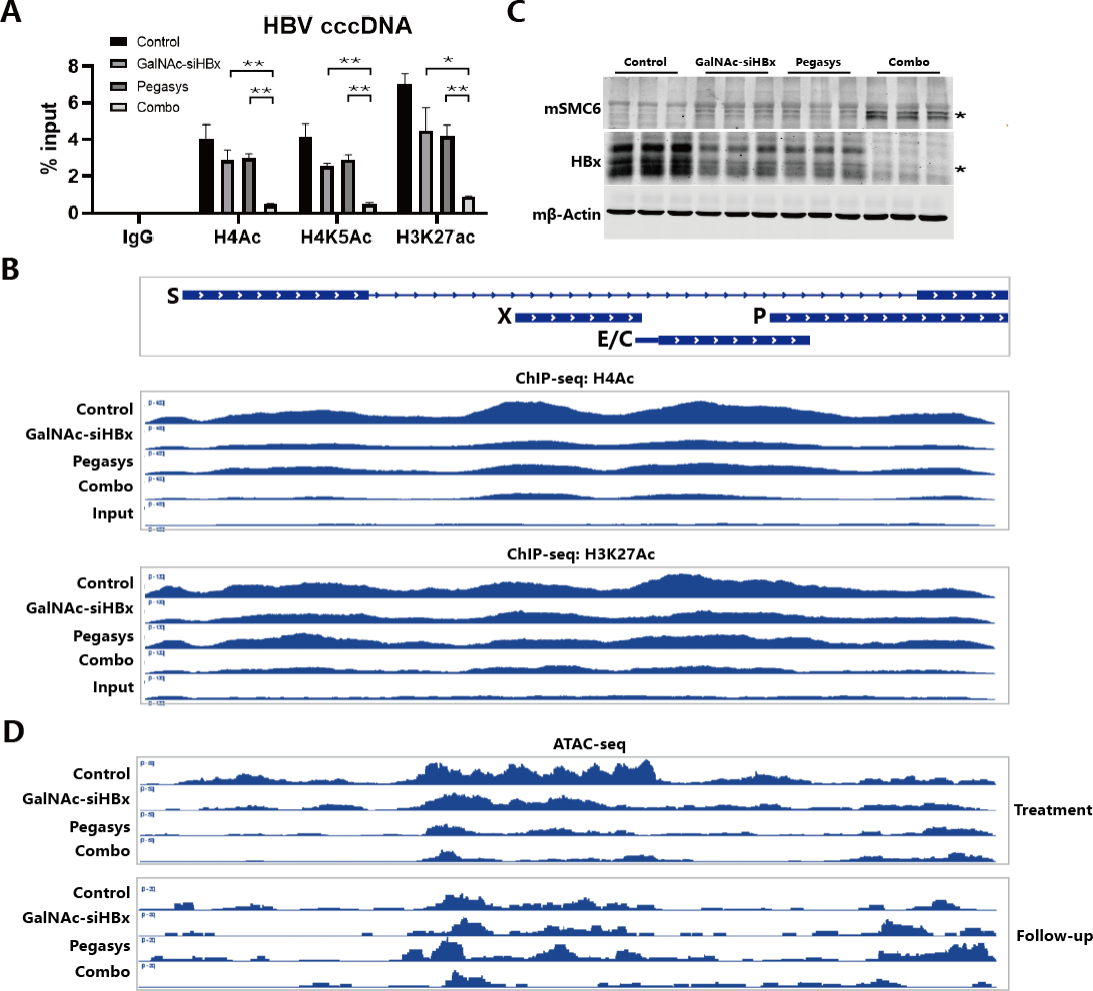
The effects of PEGIFNα2, GalNAc-siHBx, and their combination on the epigenetic modifications and accessibility of cccDNA in rcccDNA IFNAR-hEC mice. (**A**) The levels of H4Ac, H4K5Ac, and H3K27Ac modifications on intrahepatic cccDNA were determined via ChIP-qPCR analysis using relative antibodies and primers. (**B**) The levels of H4Ac and H3K27Ac modifications on intrahepatic cccDNA were determined via ChIP-seq analysis using relative antibodies. (**C**) Intrahepatic expression levels of HBx(_*_) and mSMC6(_*_) were determined via western blot analysis, and mβ-Actin was applied as loading control. (**D**) The accessibility of cccDNA in “treatment” or “Follow-up” batch was detected by ATAC-seq analysis. Data were analyzed by unpaired two-tailed Student’s *t* tests and presented as means ± SD.

We then detected the epigenetic modifications on cccDNA minichromosomes in the liver of different treatment groups of mice. ChIP-qPCR results showed that GalNAc-siHBx and Pegasys mono-treatment both partially reduced the levels of the active epigenetic modifications (H4Ac, H4K5Ac, and H3K27ac) on cccDNA, while their combination can reduce the levels of these to the greatest extent (Fig. 8A). The effect of combination treatment on the levels of H4Ac and H3K27Ac modifications on cccDNA was further confirmed by ChIP-seq (Fig. 8B). By detection of the expression of SMC5/6 in the liver of mice, we showed that the chronic rcccDNA model (the control group) led to increased HBx and reduced SMC6 expression in mouse liver, compared with the blank group mice (Fig. S14). SMC5/6 level of the control group was the lowest, while GalNAc-siHBx or Pegasys mono-treatment only slightly recovered the SMC5/6 level, while the combination treatment efficiently recovered the SMC5/6 level, highly reduced HBx level (Fig. 8C). Furthermore, we used ATAC-seq to evaluate the accessibility of cccDNA minichromosomes right at the end of treatment (treatment) and 3 weeks after stopping the drug (follow-up), showing that cccDNA minichromosomes of the combination group had the lowest accessibility over time (Fig. 8D). PEG-IFNα and GalNAc-siHBx mono- or combo-treatment displayed satisfactory safety profiles, with no evident weight loss or significant ALT/AST elevation (Fig. S15). Besides, H&E staining results indicate that there was no obvious cell infiltration in the liver of mice in all groups (Fig. S16). These results suggest that the combination of GalNac-siHBx and PEG-IFNα2 can reduce the accessibility of cccDNA, and this suppressive effect has a certain durability after stopping the treatment.

## DISCUSSION

In recent years, efforts have focused on achieving a functional cure for CHB, with the virological basis being that, although cccDNA reservoirs persist in the nuclei of infected cells, they remain transcriptionally silent (30). Observations based on liver biopsies have revealed that during the chronic HBV infection, accompanied by HBeAg seroconversion, cccDNA shows a degree of activity reduction (31), and HBsAg loss could be associated with decreased cccDNA transcription upon NA discontinuation in HBeAg-negative patients (32). In patients undergoing long-term NA treatment and those functionally cured, cccDNA levels are lowered, and the remaining cccDNA persists in a silent or low-activity state (33–35). However, the efficiency of cccDNA suppression achievable under current clinical treatment remains unsatisfactory. In recent years, treatments based on siRNA as a candidate drug for CHB have entered clinical research, showing a potent effect in reducing HBV antigens, but with a high tendency of rebound (36). Clinical trials have already used a combination of IFN and siRNA to treat chronic hepatitis B (37), but due to the difficulties in studying cccDNA, research on the effects of the combination has been mostly limited to antigen and RNA level. Our study, using a series of *in vitro* and *in vivo* models, systematically observes the effect of the combination on the degree, durability, and stability of cccDNA suppression (Table S4).

IFNα is currently a key drug for achieving hepatitis B cure and inhibits different stages of the HBV life cycle by inducing ISGs (12, 38). Previous studies have shown that IFNα can induce cccDNA degradation (39); however, this effect was not significant in our experimental system with the IFN dosage used. Notably, accumulating evidence has demonstrated that IFNα reduces the accessibility and transcriptional activity of the cccDNA minichromosome by regulating epigenetic modifications (19, 20, 22). However, such epigenetic regulation by IFNα has limited efficiency. HBx plays a vital role in the replication and pathogenesis of HBV, especially in initiating and maintaining the transcription of cccDNA. Compared to infections with wild-type HBV, infections with HBV virions lacking HBx expression produce significantly fewer HBV RNAs, and supplementing HBx restores their production (10, 40). Mechanistic studies suggest that HBx can increase active epigenetic modifications on cccDNA, reduce repressive modifications, and promote cccDNA transcription by targeting host restriction factors (6, 41, 42). Thus, siRNA targeting HBx can indirectly inhibit HBx expression and further suppress cccDNA, serving as a good complement to the broad-spectrum antiviral effects of IFNα from a mechanistic perspective. In the present study, in addition to synergistic effect, linear additive effect of the IFN and siHBx combination is significant across various concentration combinations (Fig. S3A through C). Considering the unique complementary mechanisms of the two drugs, both synergistic and additive effects should have potential therapeutic value. A recently published clinical study further supports the benefit of the combination of siRNA (VIR-2218) and pegIFN-α2a in sustained viral control (37).

A study using immunodeficient human-liver chimeric mouse models of HBV infection has shown that treatment with IFNα and siRNA can effectively suppress HBV antigens and RNAs, and both can restore the expression level of SMC5/6 in the liver, but viral markers rebound after stopping the drugs without new infection inhibition (18), suggesting a limitation of using IFN or siRNA alone. However, the extent of cccDNA repression by IFNα or siRNA was still not clear. It should be noted that the present study was conducted in HBV-infected HepG2^NTCP^ model and rcccDNA IFNAR-hEC mouse model, in which models HBV *de novo* infection barely occurs (Fig. S4). We further revealed that the combination of the two has an additive effect on cccDNA suppression, supporting their mechanistic complementarity. The relative short-half lives of siRNA were evidenced by FITC-labeled siRNAs, indicating that the persistent inhibitory effects on cccDNA might be due to epigenetic mechanisms on cccDNA. Further investigation of the stability of RISC complex-associated siRNAs may help interpreting the durable effects of siRNA. As expected, in HBV infection cell models, the combination can efficiently reduce the accessibility of cccDNA. We observed significant reductions in active epigenetic modifications on the cccDNA minichromosome (Fig. 4A). To further verify the potency of cccDNA suppression by the mono or combined use, we reactivated cccDNA using a deacetylase inhibitor (Fig. 6B and C); the results showed no significant changes in HBeAg and HBsAg levels in the combination group, while the monotherapy groups showed varying degrees of reactivation at early time point. Differences in activation and epigenetic regulation of different HBV promoters and regions may have a certain impact in this context (43, 44), and the factors mediating cccDNA silencing still require further explorations. On the mechanism of cccDNA reactivation, recent studies have shown that the repressive state of cccDNA can be activated by incoming virus through superinfection (45). Our study shows that the combination of IFNα and siRNA can effectively suppress HBV expression, which to a large extent may hinder the formation of new HBV particles and thus minimize HBV *de novo* infection. Besides, high HBV protein expression has been shown to be associated with IFN-signaling impairment (13, 46, 47), and the combined use of siRNA could have an alleviating effect on this.

HBV gene sequences integrated into the host genome are an important source of HBV antigen production (48–50). Compared to cccDNA, integrated HBV DNAs might be more difficult to clear. For HBV antigens from this source, it is generally believed that they could be cleared by activating the adaptive immune system. From this perspective, siRNA targets both cccDNA and integrated HBV DNA-derived transcripts and can open an immune intervention window by reducing the viral antigen burden (51, 52). In the combination of IFNα and siRNA, siRNA not only helps enhance the direct antiviral effect of IFNα but could also play a role in enhancing its immunomodulatory effect (data unpublished). It should be noted that no obvious cytotoxicity effect was observed during the relatively short observation period in the rcccDNA mouse model used in this study. Moreover, there is a possibility that innate immune cells or other indirect factors contributed to the synergistic effect. In an ongoing clinical study, the combination of VIR-2218 and PEG-IFNα significantly reduces HBsAg levels, showing a greater likelihood of HBsAg loss compared to monotherapy. However, the durability of this loss and the epigenetic changes of cccDNA, along with the epigenetic status of integrated HBV, requires further study. In addition, the contributions of the antiviral effect and immune activation to cccDNA silencing in different stages of treatment remain to be clarified (53, 54), which is technically challenging and would require careful analysis based on classical and more virological and immunological indicators (55).

In summary, this study comprehensively observed and clarified that the combination of IFNα and siRNA can more effectively and durably suppress cccDNA activity, which is related to the decrease in epigenetic modifications and accessibility of cccDNA minichromosomes. This deepens the understanding of the persistence and stability of HBV cccDNA modulation, providing a new perspective and scientific theoretical basis for clinically achieving a more effective cure for chronic hepatitis B through the combination therapy.

## SUPPLEMENTAL MATERIAL

Supplemental material mbio.02415-24-s0001.docx Tables S1 to S4; Fig. S1 to S16.

ASM does not own the copyrights to Supplemental Material that may be linked to, or accessed through, an article. The authors have granted ASM a non-exclusive, world-wide license to publish the Supplemental Material files. Please contact the corresponding author directly for reuse.
