# Supplementary material for: Augmented epigenetic repression of hepatitis B virus covalently closed circular DNA by interferon-α and small-interfering RNA synergy
Source: mBio. 2024 Nov 21;15(12):e02415-24. doi: 10.1128/mbio.02415-24 (PMC11633095; doi:10.1128/mbio.02415-24)
Supplement: Supplemental material — Tables S1 to S4; Fig. S1 to S16. [file mbio.02415-24-s0001.docx]

**Supplemental Material**

**Augmented Epigenetic Repression of Hepatitis B Virus Covalently Closed Circular DNA by Interferon-α and Small Interfering RNA Synergy**

Kongying Hu^a#^, Wenjing Zai^a#^, Mingzhu Xu^a^, Haiyu Wang^a,b^, Xinluo Song^a^, Chao Huang^a^, Jiangxia Liu^a^, Juan Chen^c^, Qiang Deng^a,b^, Zhenghong Yuan^a,b *^, Jieliang Chen^a*^

^a^Key Laboratory of Medical Molecular Virology (MOE/NHC/CAMS), Research Unit of Cure of Chronic Hepatitis B Virus Infection (CAMS), Shanghai Frontiers Science Center of Pathogenic Microbes and Infection, School of Basic Medical Sciences, Shanghai Medical College Fudan University, Shanghai, China.

^b^Shanghai Institute of Infectious Disease and Biosecurity, Shanghai, China.

^c^Key Laboratory of Molecular Biology of Infectious Diseases (MOE), Chongqing Medical University, Chongqing, China

^#^These authors contributed equally to this work.

^*^Correspondence to:

Prof. Zhenghong Yuan, Key Laboratory of Medical Molecular Virology, Shanghai Medical College Fudan University, Shanghai, China; [zhyuan@shmu.edu.cn](mailto:zhyuan@shmu.edu.cn)

Prof. Jieliang Chen, Key Laboratory of Medical Molecular Virology, Shanghai Medical College Fudan University, Shanghai, China; [jieliangchen@fudan.edu.cn](mailto:jieliangchen@fudan.edu.cn)

**Table S1.** Antibodies used in this study.

**Table S2.** siRNA used in this study.

**Table S3.** Primers used in this study.

**Table S4.** Summary of the efficacy of IFN and siHBx mono- or combo-treatment against HBV in cell and mouse models.

**Figure S1.** HBV infection of HepG2^NTCP^.

**Figure S2.** Antiviral effects of IFN and siHBx mono- or combo- treatment in HBV-infected HepG2^NTCP^ cells.

**Figure S3.** Synergistic antiviral effects of IFNα2 and siHBx in HBV-infected HepG2^NTCP^ cells.

**Figure S4.** The inhibitory effect of MyB on HBV entry in HepG2^NTCP^ cells.

**Figure S5.** Antiviral effects of IFN and siHBx mono- or combo-treatment on HepAD38 cells.

**Figure S6.** Antiviral effects of IFN and siHBx mono- or combo-treatment on HepG2-HBV/loxp and HepDES19 cells.

**Figure S7.** IFN and siHBx mono- or combo-treatment’s effect on cell viability of different cell types.

**Figure S8.** Analysis of the *in vitro* kinetics of siRNAs.

**Figure S9.** The relative amount of cccDNA and antiviral effects of IFN and siHBx mono- or combo-treatment on HBVcircle-transfected cells.

**Figure S10.** Antiviral effects of siHBc, siHBs and siHBx.

**Figure S11.** The effect of SMC5/6 pre-knockdown on HBV infection establishment.

**Figure S12.** The induction of ISGs in mice livers.

**Figure S13.** The effect of Pegasys and GalNAc-siHBx mono- or combo- treatment on the amount of rcccDNA in rcccDNA IFNAR-hEC mice.

**Figure S14.** Intrahepatic protein expression levels in blank and control rcccDNA IFNAR-hEC mice.

**Figure S15.** Safety profiles of PEG-IFNα and GalNAc-siHBx mono- or combo- treatment.

**Figure S16.** Histological analysis of liver sections.

**Table S1.** Antibodies used in this study.

| **Antibodies** | **Source** | **Cat No.** |
| --- | --- | --- |
| anti-histone H3 | Abcam | ab1791 |
| IgG | Abcam | ab171870 |
| H3K4me3 | Abcam | ab8580 |
| H3K27ac | Abcam | ab4729 |
| H4K5ac | Abcam | ab51997 |
| AcH3 | Millipore | 06-599 |
| AcH4 | Millipore | 06-598 |
| Nse4 | Abgent | AP9909A |
| H4Ac | Active Motif | 39925 |
| Anti-SMC6 | Abcam | ab155495 |
| β-actin | Sigma | A1978 |
| Anti-HBx | In-house |  |
| Anti-core | In-house |  |

**Table S2.** siRNA used in this study.

| **siRNA** | **Sense（5’→3’）** | **Antisense（5’→3’）** |
| --- | --- | --- |
| siHBs | 5'-CGUGGUGGACUUCUCUCAAdTdT-3' | 5'-UUGAGAGAAGUCCACCACGdTdT-3' |
| siHBx | 5'-GUUCAUGUCCUACUGUUCAdTdT-3' | 5'-UGAACAGUAGGACAUGAACdTdT-3' |
| siHBc | 5'-GCGUCGCAGAAGAUCUCAAdTdT-3' | 5'-UUGAGAUCUUCUGCGACGCdTdT-3' |
| siNC | 5'-UUCUCCGAACGUGUCACGUdTdT-3' | 5'-ACGUGACACGUUCGGAGAAdTdT-3' |
| siApoB | 5'-GUCAUCACACUGAAUACCAAUdTdT-3’ | 5'-AUUGGUAUUCAGUGUGAUGACdTdT-3’ |

**Table S3.** Primers used in this study.

| **Primers** | **Sequence** |
| --- | --- |
| cccDNA-qPCR-F | ctccccgtctgtgccttct |
| cccDNA-qPCR-R | GCCCCAAAGCCACCCAAG |
| mtDNA-F | TACCGCCATCTTCAGCAAAC |
| mtDNA-R | TAAGGGCTATCGTAGTTTTCTGG |
| HBV-pgRNA-F | GCCTTAGAGTCTCCTGAGCA |
| HBV-pgRNA-R | GAGGGAGTTCTTCTTCTAGG |
| HBV-totalRNA-F | GCTTTCACTTTCTCGCCAAC |
| HBV-totalRNA-R | GAGTTCCGCAGTATGGATCG |
| GAPDH-RNA-F | GGTATCGTGGAAGGACTCATGAC |
| GAPDH-RNA-R | ATGCCAGTGAGCTTCCCGTTCAGC |
| ChIP-cccDNA-qPCR-F | GTGCACTTCGCTTCACCTCT |
| ChIP-cccDNA-qPCR-R | AGCTTGGAGGCTTGAACAGT |
| m-IFIT3-F | ATGAGTGAGGTCAACCGGGA |
| m-IFIT3-R | TTGCACACCCTGTCTTCCAT |
| m-ISG15F | ATGGCCTGGGACCTAAAGG |
| m-ISG15R | CTGGGCAATCTGCTTCTTCAG |
| m-USP18F | ccaaaccttgaccattcacc |
| m-USP18R | atgaccaaagtcagccatcc |
| mGAPDH-RNA-F | CATCTTCCAGGAGCGAGACC |
| mGAPDH-RNA-R | CCTTTTGGCTCCACCCTTCA |
| m-mtDNA-F | GATAAACCCCGCTCTACCTCAC |
| m-mtDNA-F | GCTACACCTTGACCTAACGTTTT |
| Lenti-shSMC5-F | CCGGGCTCGAGACCTCATGCAAAGACTCGAGTCTTTGCATGAGGTCTCGAGCTTTTTG |
| Lenti-shSMC5-R | AATTCAAAAAGCTCGAGACCTCATGCAAAGACTCGAGTCTTTGCATGAGGTCTCGAGC |
| Lenti-shSMC6-F | CCGGGCGCCAGTGTGTAGAGAAAGACTCGAGTCTTTCTCTACACACTGGCGCTTTTTG |
| Lenti-shSMC6-R | AATTCAAAAAGCGCCAGTGTGTAGAGAAAGACTCGAGTCTTTCTCTACACACTGGCGC |

**Table S4.** Summary of the efficacy of IFN and siHBx mono- or combo- treatment against HBV in cell and mouse models.


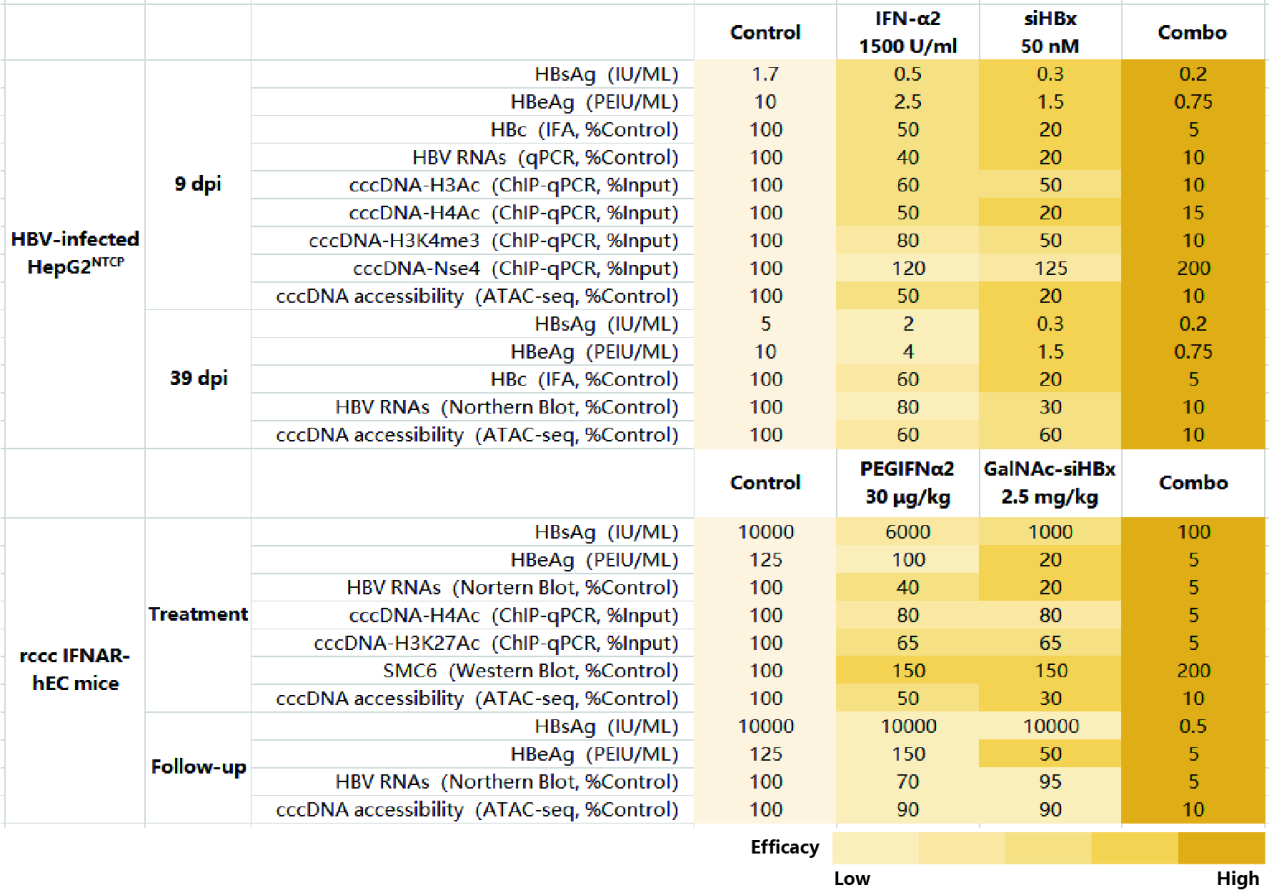


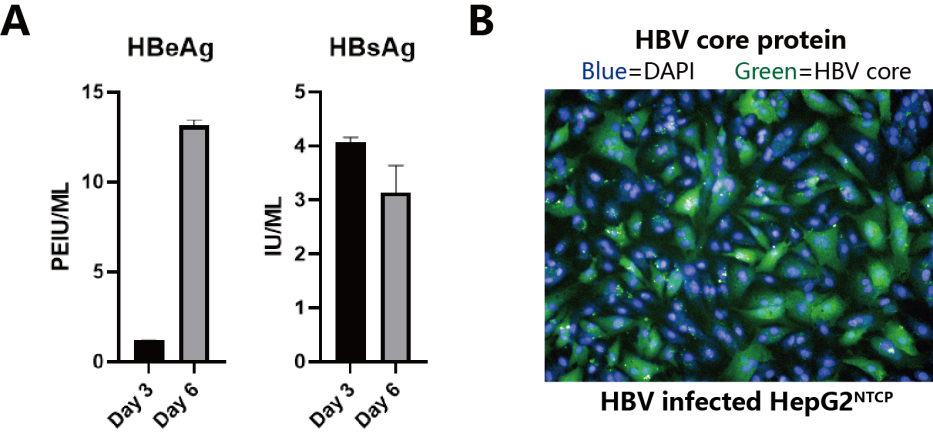


**FIG S1** HBV infection of HepG2^NTCP^. (A) HBeAg and HBsAg levels in the supernatants of HBV infected HepG2^NTCP^ cells at 3 and 6 dpi were determined by ELISA. (B) HBV core protein immunostaining of HBV infected HepG2^NTCP^ cells at 6 dpi showing an infection rate of **~**30%. HBV core (HBc) protein is shown in green, cell nuclei stained with DAPI in blue.

**
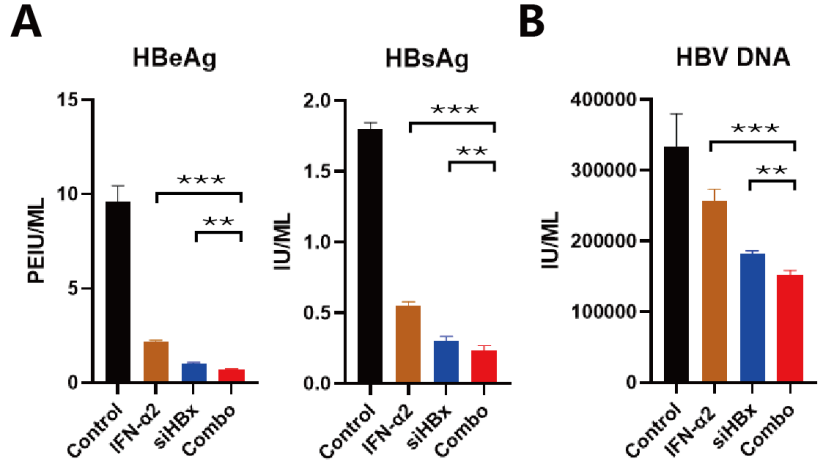
**

# FIG S2 Antiviral effects of IFN and siHBx mono- or combo- treatment in HBV-infected HepG2^NTCP^ cells. (A) HBeAg and HBsAg levels in the supernatants of HBV infected HepG2^NTCP^ cells at 9 dpi were determined by ELISA. (B) HBV DNA levels in the supernatants of HBV infected HepG2^NTCP^ cells at 9 dpi were determined by qPCR. Data were analyzed by unpaired two-tailed Student’s t tests, and presented as means±SD.

#
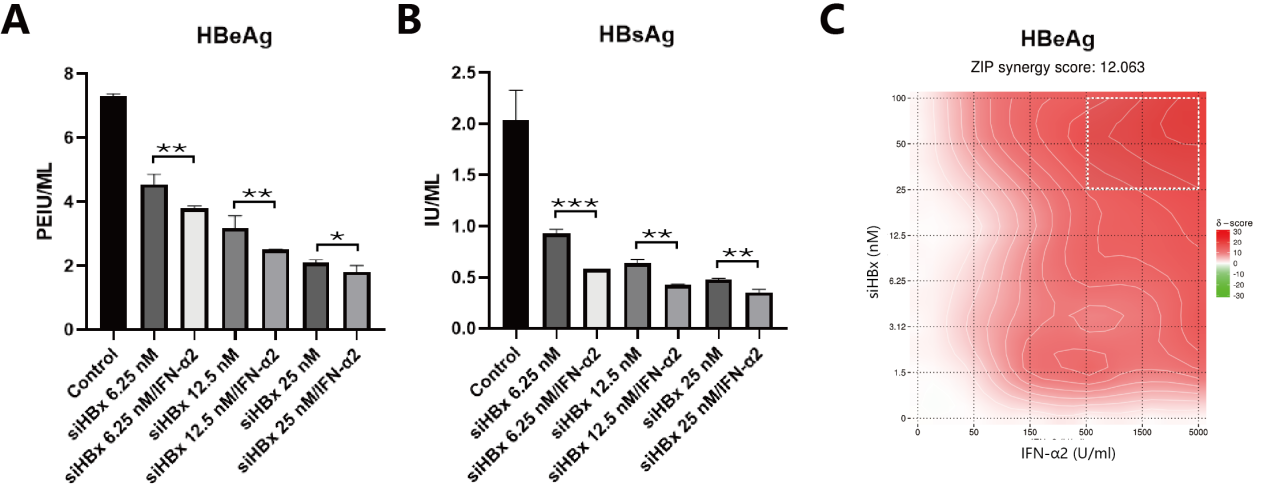
FIG S3 Synergistic antiviral effects of IFNα2 and siHBx in HBV-infected HepG2^NTCP^ cells. (A-B) HBV-infected HepG2^NTCP^ cells were treated with varying concentrations of siHBx (6.25, 12.5 and 25 nM) with or without IFNα2 (1500 U/ml) co-treatment at 6 dpi. HBeAg and HBsAg levels in the supernatants at 9 dpi were determined by ELISA. (C) HBV-infected HepG2^NTCP^ cells were treated with varying concentrations of siHBx and IFNα2 at 6 dpi. HBeAg levels in the supernatants at 9 dpi were determined by ELISA. The inhibition rates of HBeAg were calculated and imported into the software SynergyFinder. Data were analyzed by unpaired two-tailed Student’s t tests, and presented as means±SD.

**
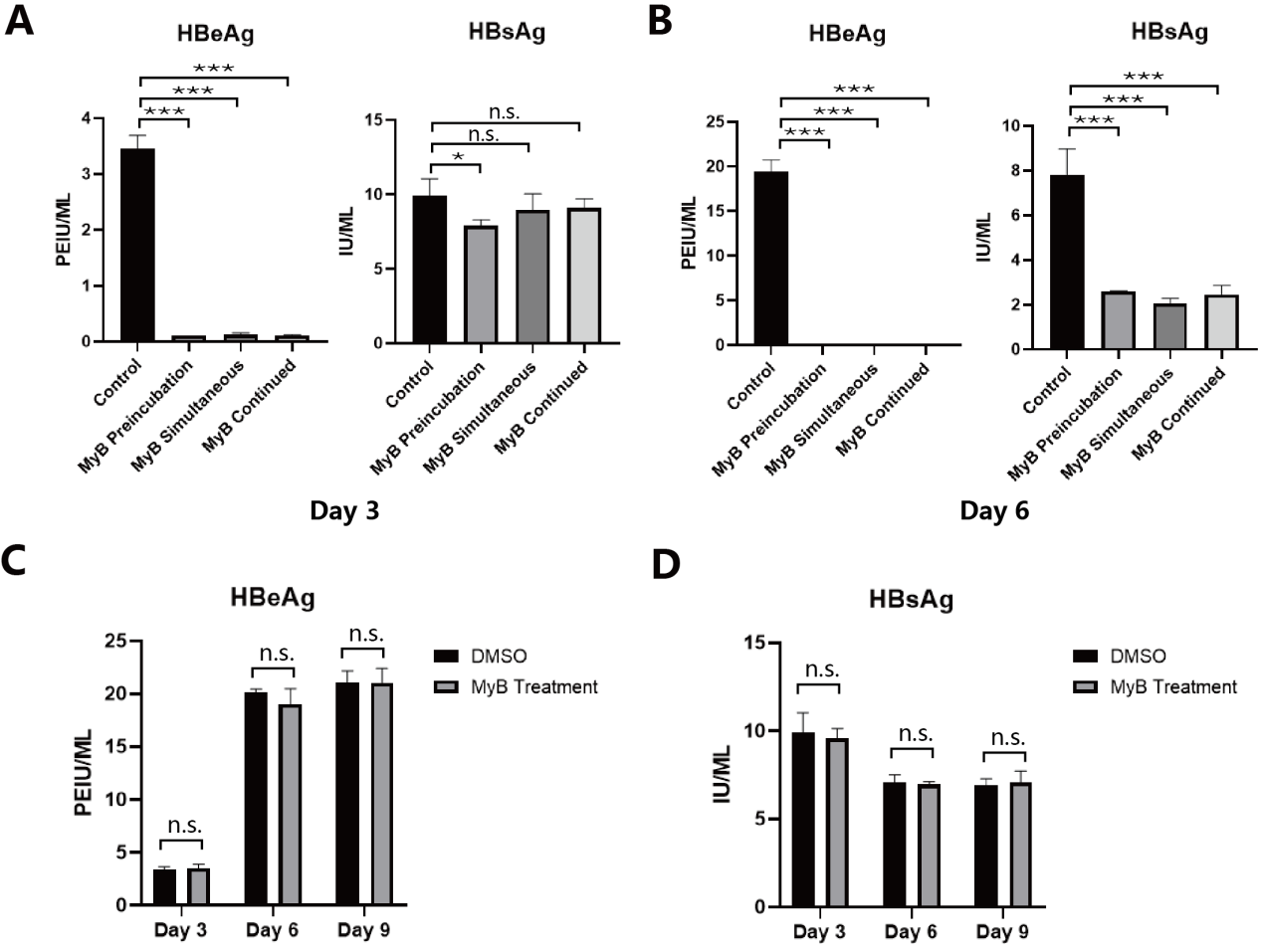
**

**FIG S4** The inhibitory effect of MyB on HBV entry in HepG2^NTCP^ cells. (A-B) MyB (400 nM) was preincubated or simultaneously incubated with HBV during infection process. Cell supernatants were collected every 3 days. HBeAg and HBsAg levels at day 3 (A) and day 6 (B) were determined by ELISA. (C) After HBV successful infection, cell supernatants were collected every 3 days. MyB (400 nM) was added in cell supernatants at day 3. HBeAg and (D) HBsAg levels were determined by ELISA. Data were analyzed by unpaired two-tailed Student’s *t* tests, and presented as means±SD.

**
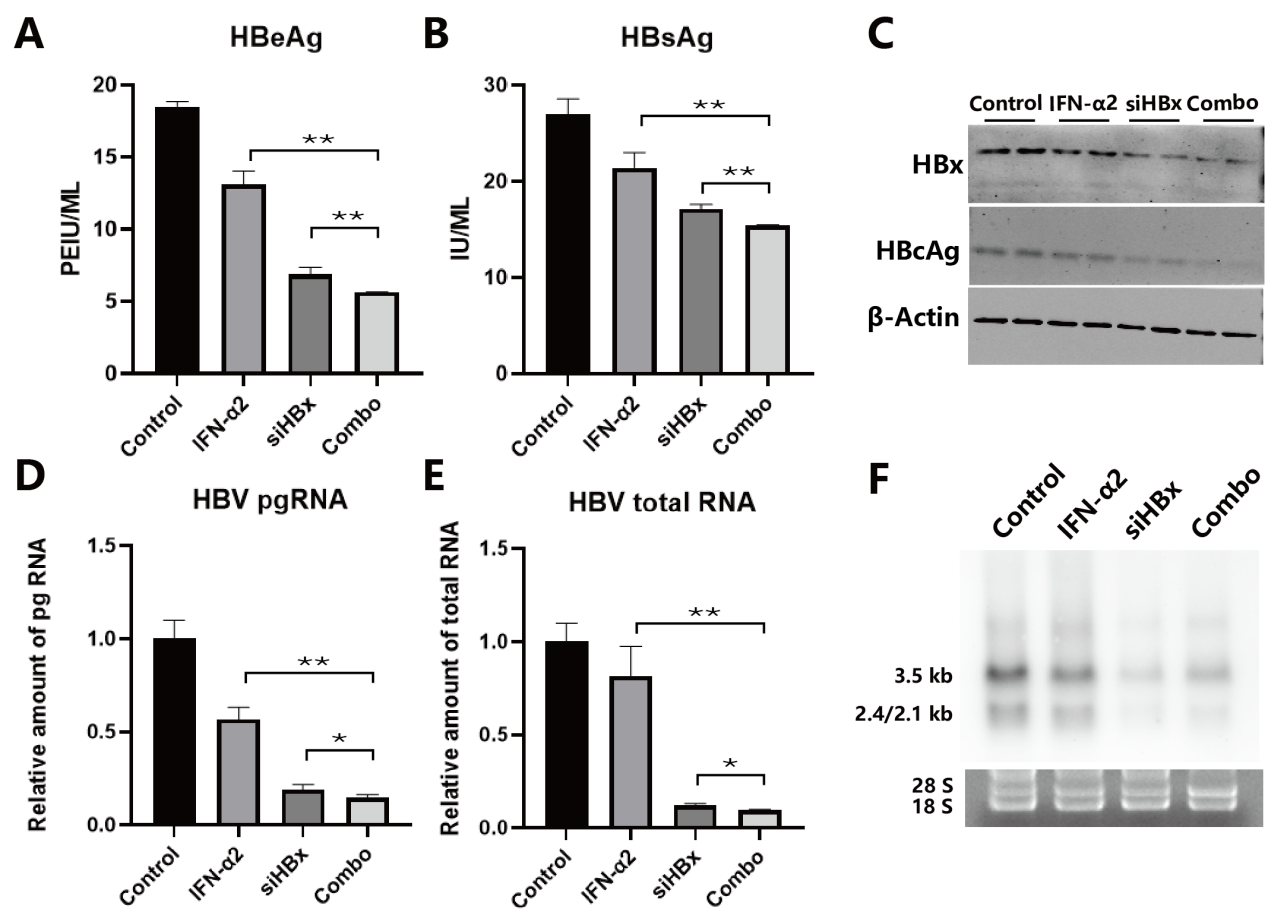
**

**FIG S5** Antiviral effects of IFN and siHBx mono- or combo-treatment on HepAD38 cells. **(**A) HepAD38 cells were treated with IFNα2 (1500 U/ml), siHBx (50 nM), or both of them. After 2 days, HBeAg and (B) HBsAg levels in the supernatants were determined by ELISA. (C) Intracellular HBx and HBcAg levels were determined via Western Blot analysis and β-Actin were applied as loading control. (D) Intracellular HBV pgRNA and (E) total RNA were determined by qPCR. (F) Intracellular HBV RNAs were determined by Northern Blot. Data were analyzed by unpaired two-tailed Student’s *t* tests, and presented as means±SD.

**
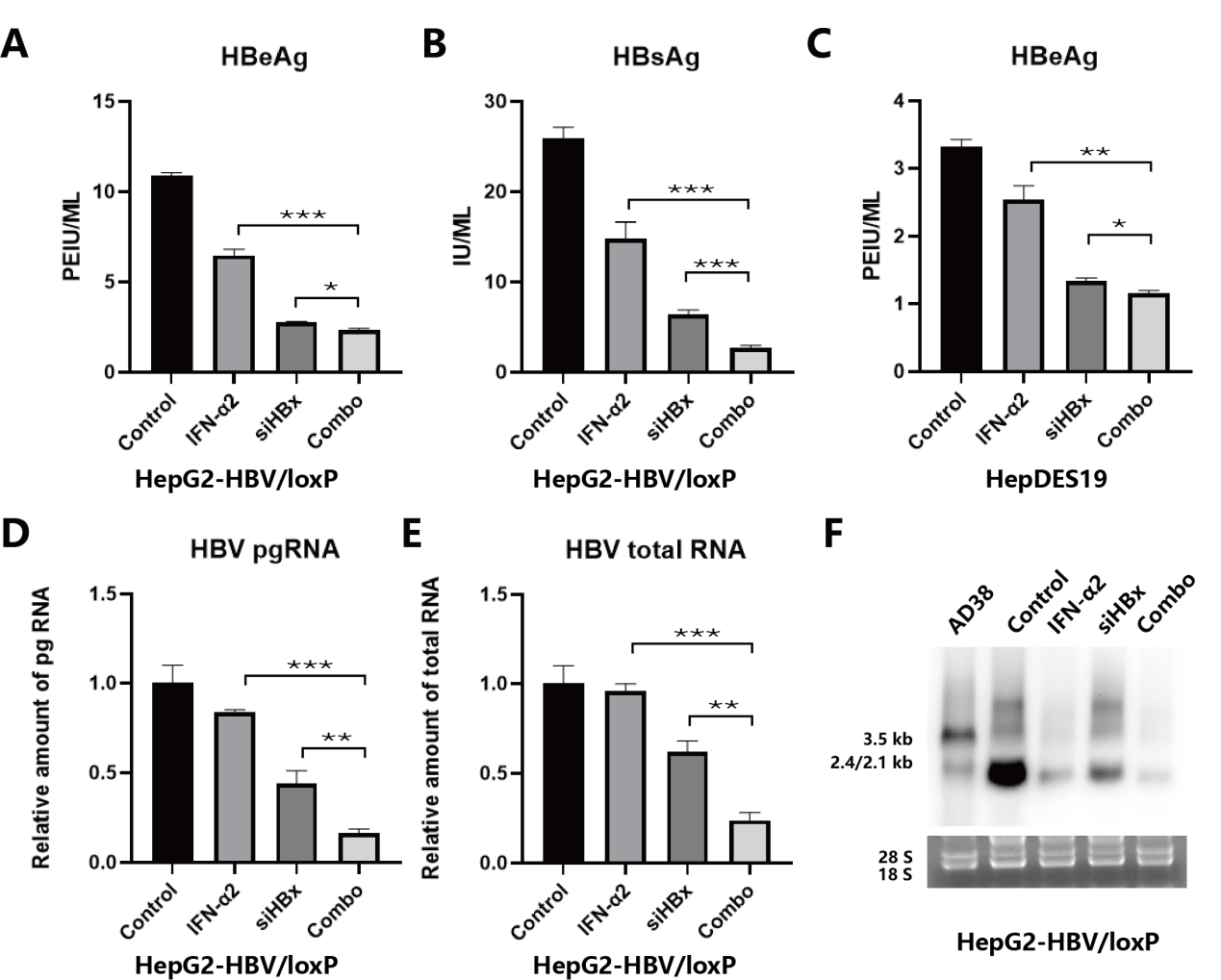
**

**FIG S6** Antiviral effects of IFN and siHBx mono- or combo-treatment on HepG2-HBV/loxp and HepDES19 cells. (A) HepG2-HBV/loxp cells were transduced with Adeno-Cre one day before treating with IFNα2 (1500 U/ml), siHBx (50 nM), or both of them. After 2 days, HBeAg and (B) HBsAg levels in the supernatants were determined by ELISA. (C) HepDES19 cells were treated with IFNα2 (1500 U/ml), siHBx (50 nM), or both of them. After 2 days, HBeAg levels in the supernatants were determined by ELISA. (D) HepG2-HBV/loxp cells were transduced with Adeno-Cre one day before treating with IFNα2 (1500 U/ml), siHBx (50 nM), or both of them. After 2 days, intracellular HBV pgRNA and (E) total RNA were determined by qPCR. (F) HepG2-HBV/loxp cells were transduced with Adeno-Cre one day before treating with IFNα2 (1500 U/ml), siHBx (50 nM), or both of them. After 2 days, intracellular HBV RNAs were determined by Northern Blot. Total RNA of Dox-off HepAD38 cells were used as Markers. Data were analyzed by unpaired two-tailed Student’s *t* tests, and presented as means±SD.

**
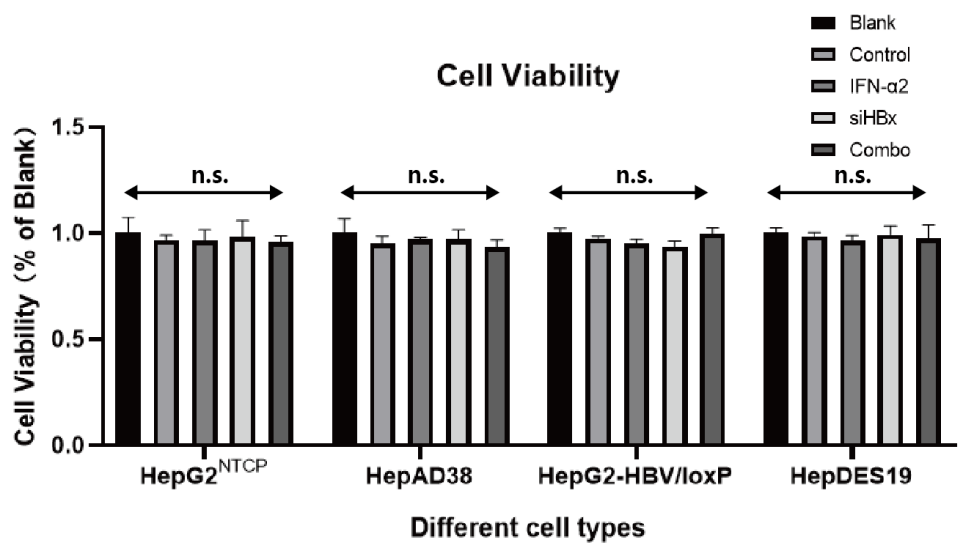
**

**FIG S7** IFN and siHBx mono- or combo-treatment’s effect on cell viability of different cell types. 3 days after IFN-α2 or siRNA treatment, cell viability analysis was conducted by CCK8. Data were analyzed by two-way analysis of variance (ANOVA) with Sidak multiple comparison correction, and presented as means±SD.

**
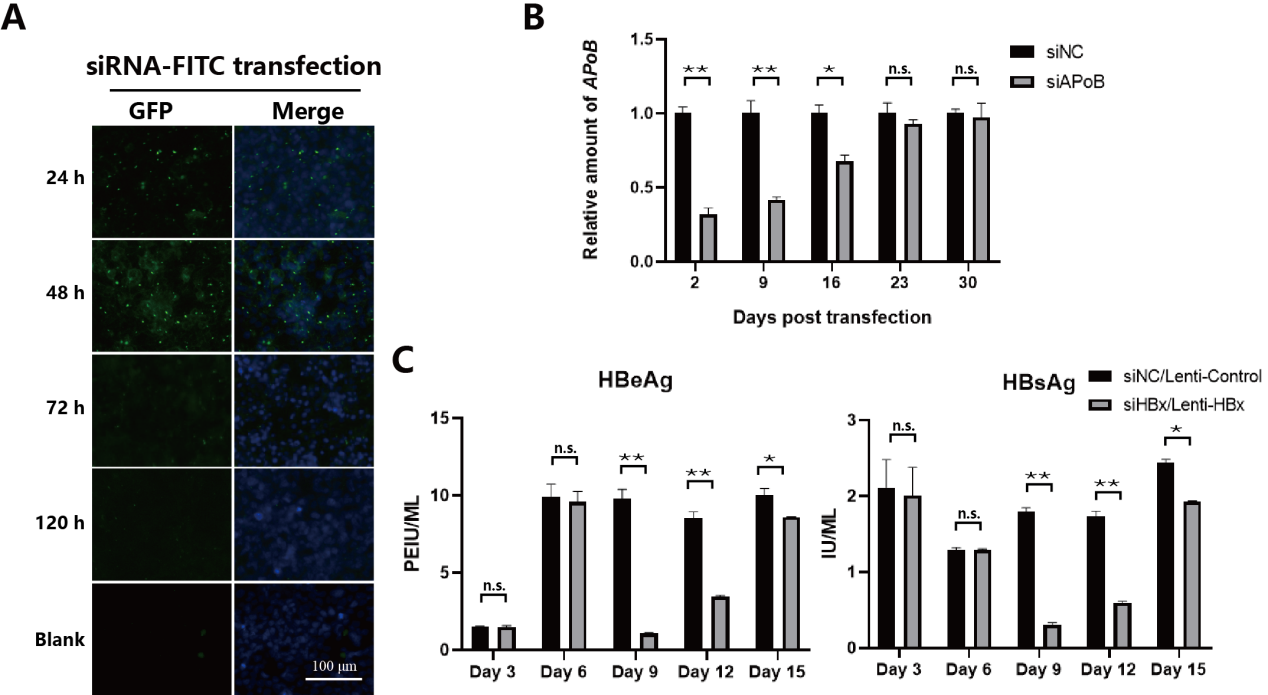
**

**FIG S8** Analysis of the *in vitro* kinetics of siRNAs and the effect of HBx trans-complementation on cccDNA activity. (A) HepG2^NTCP^ cells were transfected with siRNA-FITC, the kinetics of intracellular siRNAs were visualized at indicated time points after transfection via fluorescence microscopy. (B) HepG2^NTCP^ cells were transfected with siApoB (50 nM), relative *ApoB* mRNA levels were determined at indicated time points after transfection via qPCR analysis with specific primers. (C) HBV-infected HepG2^NTCP^ cells were treated with siHBx at 6 dpi and HBx was trans-complemented by Lenti-HBx transduction at 9 dpi. HBeAg and HBsAg levels in the supernatants were determined by ELISA at indicated time points.Data were analyzed by unpaired two-tailed Student’s t tests, and presented as means±SD.


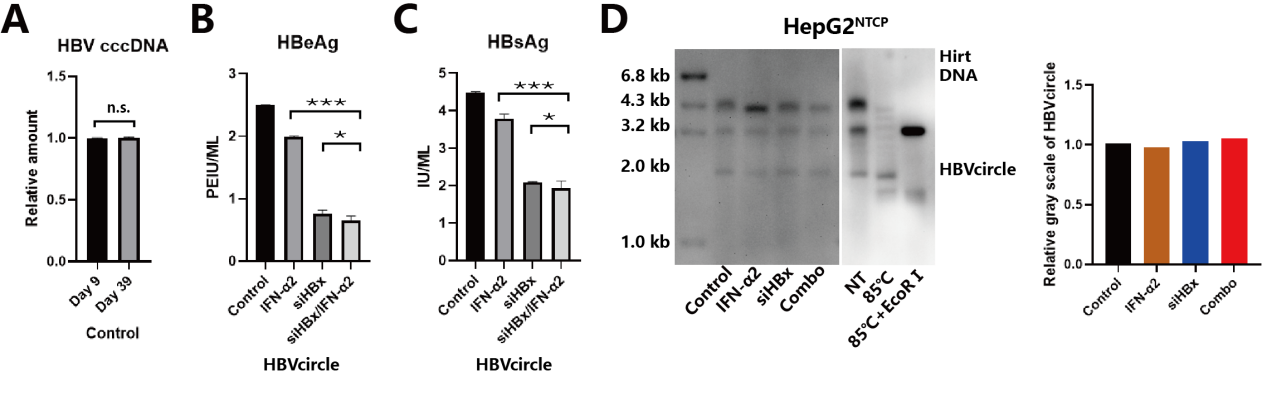
**FIG S9** The relative amount of cccDNA and antiviral effects of IFN and siHBx mono- or combo-treatment on HBVcircle-transfected cells. (A) The relative amount of cccDNA in HBV-infected HepG2^NTCP^ cells were determined via qPCR at 9 dpi and 39 dpi. (B) HepG2^NTCP^ cells were tranfected with HBVcircle, 2 days post-tranfection, cells were treated with IFNα2 (1500 U/ml), siHBx (50 nM), or both of them. After 2 days, HBeAg and (C) HBsAg levels in the supernatants were determined by ELISA. (D) HepG2^NTCP^ cells were tranfected with HBVcircle. 2 days post-tranfection, cells were treated with IFNα2 (1500 U/ml), siHBx (50 nM), or both of them. After 2 days, intracellular Hirt DNA were extracted via Hirt method, and detected by Southern Blot analysis. Data were analyzed by unpaired two-tailed Student’s *t* tests, and presented as means±SD.


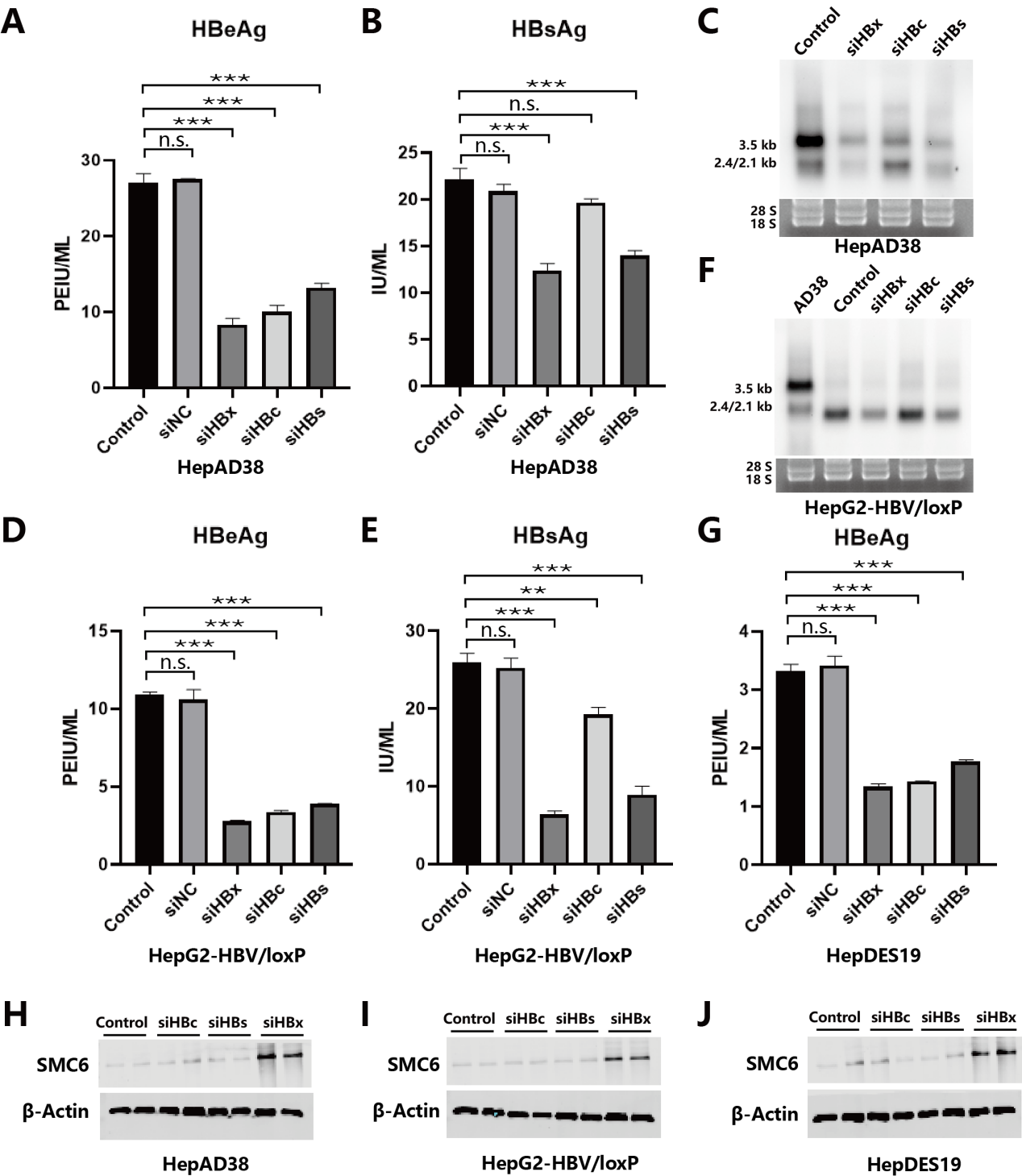
**FIG S10** Antiviral effects of siHBc, siHBs and siHBx. (A) HepAD38 cells were transfected with siNC, siHBc, siHBs or siHBx. After 2 days, HBeAg and (B) HBsAg levels in the supernatants were determined by ELISA. (C) Intracellular HBV RNAs were determined by Northern Blot. (D) HepG2-HBV/loxp cells were transfected with siNC, siHBc, siHBs or siHBx. After 2 days, HBeAg and (E) HBsAg levels in the supernatants were determined by ELISA. (F) Intracellular HBV RNAs were determined by Northern Blot. (G) HepDES19 cells were transfected with siNC, siHBc, siHBs or siHBx. After 2 days, HBeAg levels in the supernatants were determined by ELISA. (H) HepAD38, (I) HepG2-HBV/loxp and (J) HepDES19 cells were transfected with siHBc, siHBs or siHBx. Intracellular SMC6 levels were determined via Western Blot analysis and β-Actin were applied as loading control. Data were analyzed by unpaired two-tailed Student’s *t* tests, and presented as means±SD.


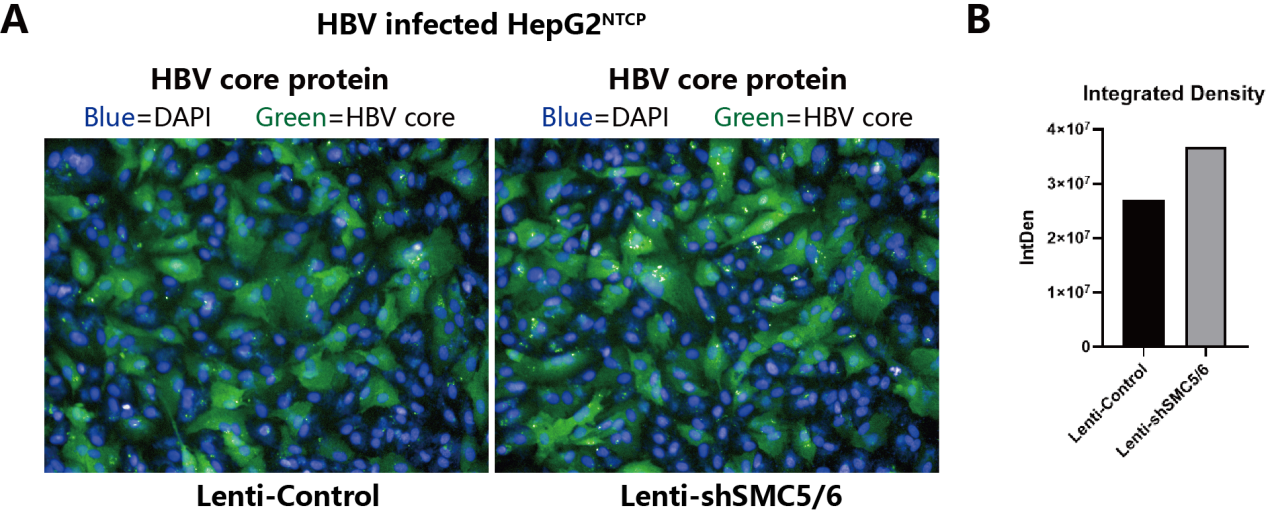


**FIG S11** The effect of SMC5/6 pre-knockdown on HBV infection establishment. (A) HepG2^NTCP^ cells were transduced with Lenti-Control or Lenti-shSMC5/6 lentivirus 1 day before infected with HBV and intracellular HBc levels at 6 dpi were determined via immunofluorescence assay. (B) The integrated density of cell immunofluorescence was measured by ImageJ.


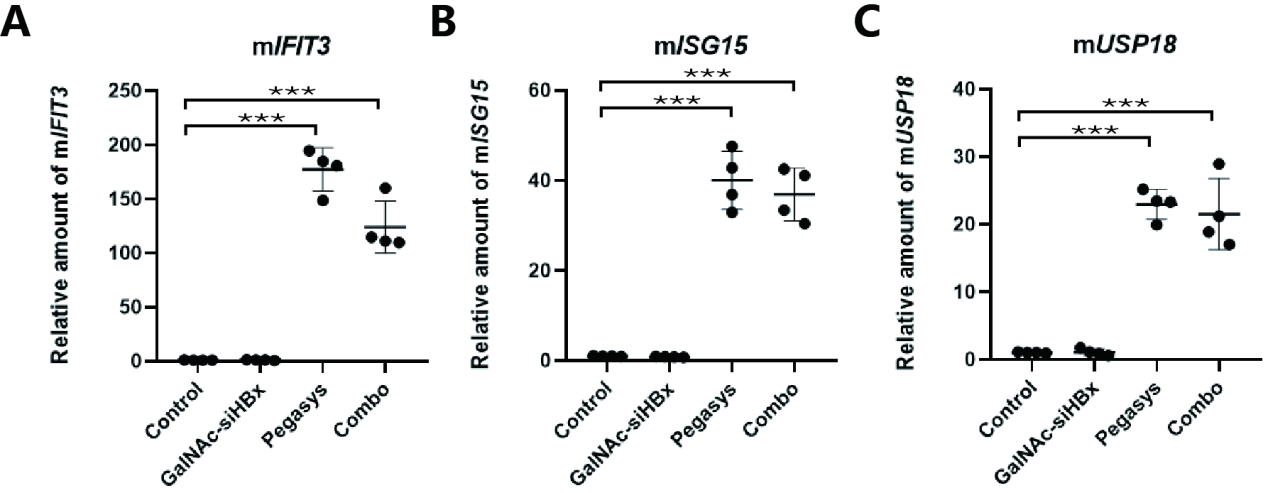


**FIG S12** The induction of ISGs in mice livers. (A-C) Six hours after the last dose of Pegasys, mice in Pegasys and combination groups in “Treatment” batch (n=4) were sacrificed. Total liver RNAs were extracted, reverse-transcripted, and applied for qPCR analysis with ISGs primers. Data were analyzed by unpaired two-tailed Student’s *t* tests, and presented as means±SD.


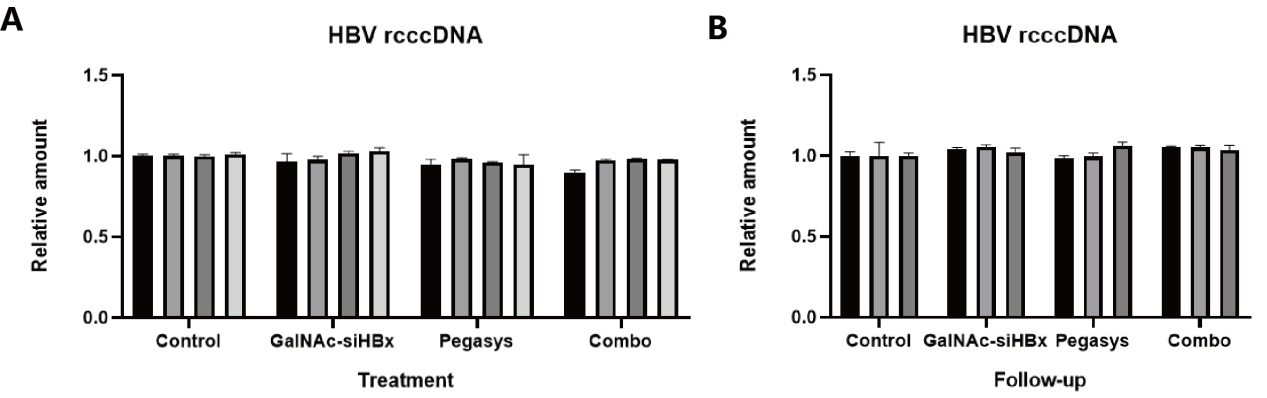


**FIG S13** The effect of Pegasys and GalNAc-siHBx mono- or combo- treatment on the amount of rcccDNA in rcccDNA IFNAR-hEC mice. Intrahepatic rcccDNA levels were measured via qPCR.


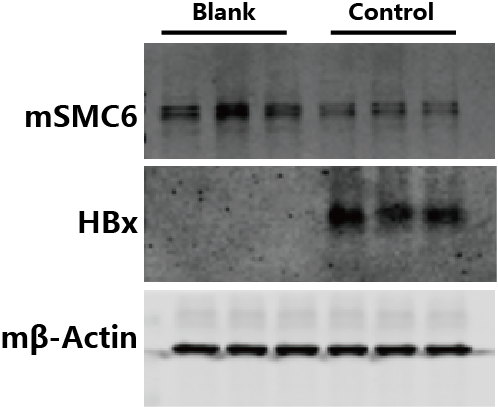


**FIG S14** Intrahepatic protein expression levels in blank and control rcccDNA IFNAR-hEC mice. Intrahepatic expression levels of HBx, mSMC6 were determined via Western Blot analysis and mβ-Actin were applied as loading control.


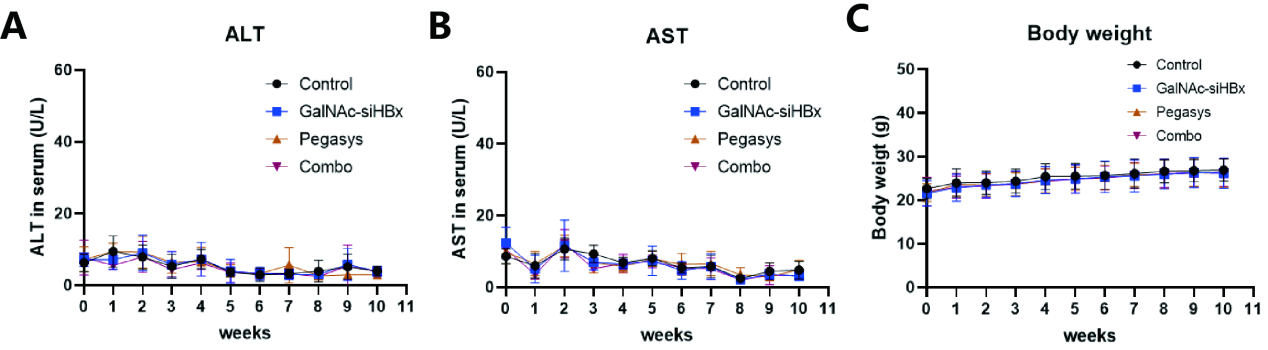


**FIG S15** Safety profiles of PEG-IFNα and GalNAc-siHBx mono- or combo- treatment. **(**A) Blood was sampled at indicated time points and were applied for ALT and (B) AST analysis. (C) The body weight of mice of different groups were displayed.


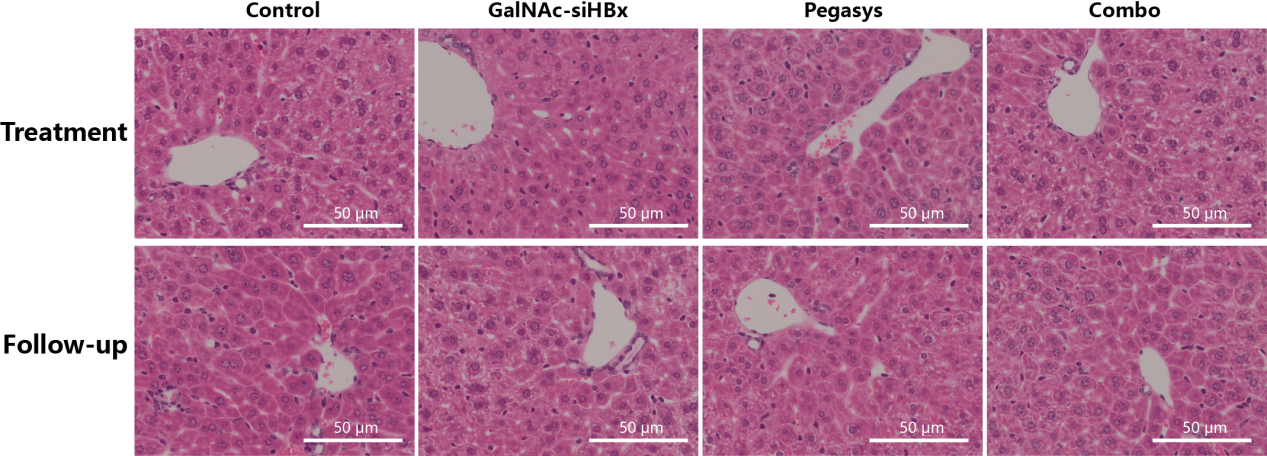


**FIG S16** Histological analysis of liver sections. Mice of the “Treatment” and the “Follow-up” batches were sacrificed at the end of observation. Liver sections were embeded in paraffin and applied for H&E staining. Representative images from different batches were shown. Scale bar indicates 50 μm.
